# Characterization of a *Haematococcus pluvialis* Diacylglycerol Acyltransferase 1 and Its Potential in Unsaturated Fatty Acid-Rich Triacylglycerol Production

**DOI:** 10.3389/fpls.2021.771300

**Published:** 2021-12-07

**Authors:** Hongli Cui, Wenxin Xu, Xiaoli Zhu, Chunchao Zhao, Yulin Cui, Chunli Ji, Chunhui Zhang, Jinai Xue, Song Qin, Xiaoyun Jia, Runzhi Li

**Affiliations:** ^1^College of Agriculture, Institute of Molecular Agriculture and Bioenergy, Shanxi Agricultural University, Taigu, China; ^2^College of Plant Protection, Shanxi Agricultural University, Taigu, China; ^3^Key Laboratory of Coastal Biology and Biological Resource Utilization, Yantai Institute of Coastal Zone Research, Chinese Academy of Sciences, Yantai, China; ^4^College of Life Sciences, Shanxi Agricultural University, Taigu, China

**Keywords:** *Haematococcus pluvialis*, type-I diacylglycerol acyltransferase, function characterization, esterified astaxanthin, triacylglycerol, genetic engineering

## Abstract

The unicellular green alga *Haematococcus pluvialis* has been recognized as an industry strain to produce simultaneously esterified astaxanthin (EAST) and triacylglycerol (TAG) under stress induction. It is necessary to identify the key enzymes involving in synergistic accumulation of EAST and TAG in *H. pluvialis*. In this study, a novel diacylglycerol acyltransferase 1 was systematically characterized by *in vivo* and *in silico* assays. The upregulated expression of *HpDGAT1* gene was positively associated with the significant increase of TAG and EAST contents under stress conditions. Functional complementation by overexpressing *HpDGAT1* in a TAG-deficient yeast strain H1246 revealed that HpDGAT1 could restore TAG biosynthesis and exhibited a high substrate preference for monounsaturated fatty acyl-CoAs (MUFAs) and polyunsaturated fatty acyl-CoAs (PUFAs). Notably, heterogeneous expression of *HpDGAT1* in *Chlamydomonas reinhardtii* and *Arabidopsis thaliana* resulted in a significant enhancement of total oils and concurrently a high accumulation of MUFAs- and PUFAs-rich TAGs. Furthermore, molecular docking analysis indicated that HpDGAT1 contained AST-binding sites. These findings evidence a possible dual-function role for HpDGAT1 involving in TAG and EAST synthesis, demonstrating that it is a potential target gene to enrich AST accumulation in this alga and to design oil production in both commercial algae and oil crops.

## Introduction

Microalgae have been recognized as the promising biological resource for commercial production of oil, healthy food, aquacultural feed, pharmaceuticals, biofuels, and bio-based chemicals (Durrett et al., 2008; Hu et al., 2008; Xin et al., 2019). Particularly, a number of oleaginous microalgae, just like oilseed plants, have been employed to produce high value-added oils although the molecular mechanism underlying lipid biosynthesis and oil accumulation in these microalgae is not elucidated in detail yet (Durrett et al., 2008; Hu et al., 2008). In order to further improve oil yield and quality, particularly for production of the designed lipids using microalgae, increasing efforts are focusing on the functional characterization of the key enzymes in fatty acid and oil biosynthesis pathways as well as corresponding regulatory networks (Xue et al., 2015; Goncalves et al., 2016; Lenka et al., 2016).

The known studies demonstrate that triacylglycerols (TAGs) are the predominant forms of storage lipid in various organisms including higher plants and microalgae. TAGs are normally assembled in endoplasmic reticulum (ER) and then deposited in oil bodies (OBs) or lipid droplets (LDs) in cells. Two pathways are found to control TAG biosynthesis in cells, namely, acyl-CoA-dependent and acyl-CoA-independent pathways (Xu et al., 2018). In the acyl-CoA-dependent pathway, diacylglycerol acyltransferase (DGAT) catalyzing the finally committed step of TAG assembly has been used as the core target for manipulating TAG production in a number of organisms (Lung and Weselake, 2006; Liu et al., 2012; Jin and Jiang, 2015; Xu et al., 2018). Currently, four types of DGATs have been identified, including the membrane-bound DGAT1 and DGAT2 with low sequence similarity between them, the cytosol-soluble DGAT3, and the dual-function of WS/DGAT having both wax ester and TAG biosynthesis activities (Xu et al., 2018).

With a great progress made in DGATs in higher plants, a growing interest has been focused on DGATs from microalgae since algal DGATs exhibited diversity in protein structure and functions as well as the highlighted significances in biotechnology for increasing TAG production in both oilseed plants and oleaginous microalgae (Xu et al., 2018). It was previously reported that different types of algal DGATs played distinct roles in TAG biosynthesis. Generally, one or two isoforms of DGAT1 enzymes function critically in determining carbon flux into biosynthesis of TAGs enriched with normal fatty acids (FAs), whereas multiple DGAT2 members play important roles in formation of the TAG-containing unusual FAs (Xu et al., 2018; Zienkiewicz et al., 2018). Multiple isoforms were identified for DGAT1, DGAT2, and DGAT3 in a number of microalgae. For example, *Chlamydomonas reinhardtii* was detected to have one *DGAT1*, five *DGAT2*, and one *DGAT3* genes (Liu et al., 2016; Bagnato et al., 2017)*. Nannochloropsis oceanica* contained two *DGAT1* and eleven *DGAT2* genes (Li et al., 2016; Wei et al., 2017; Zienkiewicz et al., 2017). An astaxanthin-producing *Chlorella zofingiensis* was tested to possess two *DGAT1* and eight *DGAT2* genes (Mao et al., 2019; Xu et al., 2019). Moreover, *Phaeodactylum tricornutum* was identified to have four types of *DGAT* genes (a *DGAT1*, four *DGAT2s*, a *DGAT3*, and a dual-function *WS/DGAT*; Guihéneuf et al., 2011; Gong et al., 2013; Cui et al., 2018). Taken together, these previous reports indicated that diverse algal *DGAT* genes showed a large difference in their transcription levels, enzymatic activities of the encode proteins, and engineering potentials in lipid production (Guihéneuf et al., 2011; Gong et al., 2013; Li et al., 2016; Liu et al., 2016; Bagnato et al., 2017; Guo et al., 2017; Klaitong et al., 2017; Wei et al., 2017; Xin et al., 2017, 2019; Zienkiewicz et al., 2017; Cui et al., 2018; Mao et al., 2019; Xu et al., 2019). Therefore, it is much required to excavate additional novel DGATs from other industrially important microalgae like *Haematococcus pluvialis*, a high producer of astaxanthin (AST).

*Haematococcus pluvialis* is well acknowledged as the ideal natural producer of AST that is a kind of keto-carotenoids with super antioxidant activity (Lorenz and Cysewski, 2000). Furthermore, this microalga is able to produce a large amount of oils (Chen et al., 2015; Bilbao et al., 2020). Particularly, under stress inductions, a significant association is detected for TAG and AST accumulations in *H. pluvialis* (Chen et al., 2015). Such concurrence could be explained by the finding that ASTs in *H. pluvialis* are deposited in oil bodies enriched with the bulk of TAGs and the predominant forms of mono-estered AST (M-EAST, 70%) and di-estered AST (D-EAST, 25%) (Miao et al., 2006; Yang et al., 2021). Moreover, AST accumulation may depend on TAG assembly since the addition of DGAT-specific inhibitors in algal medium resulted in considerable reduction for both compounds (Chen et al., 2015). Based on these, it has been hypothesized that as the committed enzyme in TAG biosynthesis, DGAT may be the key candidate for AST esterification in *H. pluvialis* (Chen et al., 2015; Ma et al., 2018; Zhao et al., 2020). Thus, further functional characterization of *H. pluvialis* DGATs will generate new knowledge to elucidate the mechanism of AST esterification. If a DGAT responsible for AST esterification is identified, such DGAT can be employed as the target in genetic engineering to largely increase the production of both valued TAGs and ASTs in the interested hosts.

To date, one *DGAT1* and five *DGAT2* (*HpDGAT2A*, *HpDGAT2B*, *HpDGAT2C*, *HpDGAT2D*, and *HpDGAT2E*) genes have been identified in *H. pluvialis* (Ma et al., 2018; Cui et al., 2021). Except for HpDGAT2C (HpDGTT2), the other four HpDGAT2s displayed the enzymatic activity to restore TAG biosynthesis in a TAG-deficient yeast strain when heterologously overexpressed in the host (Nguyen et al., 2020; Cui et al., 2021; Ma et al., 2021). Furthermore, two *DGAT2* genes (*HpDGTT2* and *HpDGAT2D*) were examined as the potential targets in genetic engineering for valued oil production in a number of microalgae and higher plants (Cui et al., 2021; Ma et al., 2021). However, none of DGAT1 was functionally examined in *H. pluvialis.* It remains unknown whether HpDGAT1 or other enzymes are responsible for AST esterification.

In this study, a novel HpDGAT1-encoding gene was isolated from *H. pluvialis*, followed by bioinformatics analysis on the physico-chemical features of this protein. The *HpDGAT1* expression together with AST and TAG accumulations were detected to be significantly increased under stress conditions of high light and nitrogen deficiency. The enzymatic activity and substrate specificity of HpDGAT1 were extensively characterized using functional complementation assay in the yeast mutant H1246 and exogenous fatty acids feeding experiments. The function of HpDGAT1 in AST esterification was further predicted by *in silico* molecular docking assay. Finally, the biotechnology applications of *HpDGAT1* gene in improving TAG yield and quality were investigated by heterologous expression of this gene in both model microalga *Chlamydomonas reinhardtii* and higher plant *Arabidopsis thaliana*. The present data provide new insights into understanding of HpDGAT1’s functions in both TAG and EAST biosynthesis in *H. pluvialis*.

## Materials and Methods

### Strain and Growth Conditions

*Haematococcus pluvialis* FACHB-712 was obtained from Freshwater Algae Culture Collection at the Institute of Hydrobiology, CAS, China. Algal cells were cultivated in 100 ml of BBM medium in a 250-mL Erlenmeyer flask under normal growth conditions (23±1°C, light intensity of 25 μmolm^−2^ s^−1^, and a diurnal cycle of 12 h light/12 h dark). The algal cultures at the later exponentially growing stage (biomass ~200 mg L^−1^) were used for high light with blue (HL-B), high light with white (HL-W), nitrogen deficient (ND), high light with blue coupled nitrogen deficient (HL-B-ND), and high light with white coupled nitrogen deficient (HL-W-ND) treatments, respectively, according to our previous descriptions (Cui et al., 2021).

### Molecular Cloning, Sequence Analysis, and Expression Profiling of *HpDGAT1* Gene

Total RNA extraction, quantification, and the first-strand cDNA synthesis were conducted using EasySpin RNA Extraction Kit (Aidlab Biotech, China), NanoDrop 2000c (Thermo Scientific, United States), and PrimeScript^®^ RT Enzyme Mix I Kit (TaKaRa DRR047A, China), respectively. The local BLAST program based on the *H. pluvialis* transcriptome database together with RACEs methods were employed to obtain the full-length cDNA sequence of *HpDGAT1*. The putative HpDGAT1 and other annotated DGATs were listed in Supplementary Table S1. All primers were listed in Supplementary Table S2. The physico-chemical properties of HpDGAT1 protein were predicted in ExPASy (Gasteiger et al., 2003), including molecular weight (Mw), isoelectronic point (pI), sub-cellular localization, signal peptides (SP), chloroplast transfer peptides (CTP), trans-membrane regions (TM), and phosphorylation sites (Phos). HpDGAT1 and other DGATs from plants and microalgae were aligned using ClustalX (Thompson et al., 2002). Maximum likelihood tree was constructed using PhyML (Guindon et al., 2010). The transcription expression of *HpDGAT1* was examined using qRT-PCR running on a 7500 Fast Real-Time PCR System (Applied Biosystems, Waltham, MA, United States) with SYBR Green PCR Master Mix (Invitrogen) Kit as described in our previous study (Cui et al., 2021).

### Functional Characterization of *HpDGAT1* in Yeast Mutant Strain H1246

For yeast (*Saccharomyces cerevisiae*) mutant H1246, four genes (*DGA1*, *LRO1*, *ARE1*, and *ARE2*) contributing to TAG synthesis was knocked out, and thus, such mutant is TAG-deficient. This H1246 was used to investigate the function of HpDGAT1. The ORF of *HpDGAT1* was cloned into the yeast expression vector pYES2.0 (Invitrogen) to construct the recombinant pYES2.0-*HpDGAT1* vector. After sequence confirmation, the recombinant vector was transformed into both wild-type *S. cerevisiae* INVSc1 and mutant H1246 with the S.c. EasyComp Transformation Kit (Invitrogen, United States). PCR was used to verify the presence of pYES2.0-*HpDGAT1* vector in the yeast cells. The expression of *HpDGAT1* gene in the yeast strain was induced by 2% (w/v) galactose SD/−ura medium, followed by qRT-PCR analysis on the transcription level of *HpDGAT1*. The supplementation of each FA (C18:2n6, C18:3n3, C18:3n6, and C20:4n6) was added to the culture to a final concentration of 100 μm, respectively, at the beginning of galactose induction as described by Siloto et al. (2009). Briefly, FAs were dissolved in ethanol at 0.5 M. FAs solutions were diluted in a warm medium containing 0.01% (v/v) Tergitol NP-40 (Sigma-Aldrich, St. Louis, MO, United States) immediately before yeast inoculation. Control experiments without FAs contained the same volume of ethanol and 0.01% (v/v) of Tergitol NP-40. Yeast samples at an OD600 of 2.5 were harvested, and lipids were extracted for TAG separation by TLC and FA composition analysis by GC. Briefly, 50 mg of yeast cells was used to extract total lipids (Bligh and Dyer, 1959). Then, TAGs were separated by thin-layer chromatography (TLC) methods as descripted in previous study (Liu et al., 2019). Finally, TAGs were trans-esterified with 5% H_2_SO_4_ in methanol at 85°C for 1 h and the fatty acid methyl esters (FAMEs) were analyzed by an Agilent GC equipped with a flame ionization detector (FID) and a capillary column (HP-88100 m × 0.25 mm × 0.2 mm) with an appropriate add amount of C17:0 FAME (Sigma) as an internal standard (Liu et al., 2019; Cui et al., 2021).

### Yeast Microsome Preparation and *in vitro* Acyl-CoA Substrate Specificity Assay

Yeast microsome was prepared as described by Siloto et al. (2009) and Liu et al. (2016). Briefly, induced yeast cultures were collected at 3,000 g for 5 min and washed twice with 1 ml of buffer containing 20 mm Tris–HCl pH 7.5, 10 mm MgCl_2_, 1 mm EDTA, 5% (v/v) glycerol, 300 mm ammonium sulfate, and 2 mm dithiothreitol and lysed by a bead beater (Biospec, Bartlesville, United States) using 0.5 mm glass beads. Then, cell debris was removed from the suspension by centrifugation at 10,000 g for 10 min at 4°C, and the supernatant was recovered by centrifuging further at 100,000 g for 1 h at 4°C. The resulting microsomal membrane pellets were resuspended in microsomal storage buffer containing 50 mm Tris–HCl, pH 7.5, 10% (v/v) glycerol with a protein concentration of 10 μg μL^−1^ for immediate use or storage at −80°C. The *in vitro* acyl-CoA substrate specificity of HpDGAT1 assay was performed in a 200 μl volume of mixture containing 40 μg microsomal membrane protein, 25 mm sucrose, 0.1 mm EDTA, 15 mm Tris–HCl pH 7.5, 125 μgml^−1^ BSA (FA-free), 250 μm acyl CoA, and 250 μm DAG (eukaryotic type C16:0/C18:1n9 DAG and prokaryotic type C18:1n9/C16:0 DAG were used as the acyl acceptor) as described method by Liu et al. (2016). The DAGs were purchased from Larodan Fine Chemicals.^1^ The other lipid standards were purchased from Avanti Polar Lipids.^2^ The reactions were incubated at 30°C for 1 h, and the lipids were extracted and detected as described above. The microsome fraction alone and the microsome fraction with different DAGs were used as control. The assays were performed in three technical replicates.

### Genetic Engineering of *HpDGAT1* in Microalgae and Higher Plants

For genetic engineering in microalgae, the ORF of *HpDGAT1* was optimized for codon preference according to microalga *C. reinhardtii* CC849 and then cloned into the algal nuclear transformation expression vector pDB124 with the *Pml* I and *Bmt* I sites in the form of *HpDGAT1*-*His* fusion gene as described in our previous study (Cui et al., 2021). Briefly, after sequence confirmation, the resulting pDB124-*HpDGAT1*-*His* vector was linearized by *Xba* I digestion, and subsequently transformed into the *C. reinhardtii* CC849 strain *via* the glass beads method. Algal transformants were selected on Tris-acetate-phosphate (TAP) plates with 10 μgml^−1^ bleomycin (Sigma-Aldrich). *C. reinhardtii* CC849 cells at the later exponentially growth stage (biomass ~420 mg L^−1^) were treated for ND stress using methods described above. The integration of *HpDGAT1* gene into the *Chlamydomonas* genome was verified by genomic PCR. Afterward, the transcription and protein expression levels of *HpDGAT1* were verified by qRT-PCR and Western blotting using his-tagged antibodies, respectively, according to the method reported previously (Cui et al., 2021).

For genetic engineering in higher plants, the codon optimized ORF of *HpDGAT1* was cloned into *Nco* I and *Spe* I sites of pCAMBIA1303 to yield pCAMBIA1303-*HpDGAT1* vector under the expression cassette of CaMV 35S promoter and NOS terminator. This recombinant plant expression vector was transferred into *Agrobacterium tumefaciens* strain GV3101 by the freeze–thaw method (Holsters et al., 1978). After verification, the positive clones were cultured in liquid medium and then used to infect *Arabidopsis thaliana* by vacuum infiltration (Clough and Bent, 1998). T1 generation seeds were selected on hygromycin (50 mg L^−1^), and then, the selected transformed plants were transferred to soil in pot for growing to maturation. T2 transgenic *A. thaliana* lines (*At-HpDGAT1-4*, *At-HpDGAT1-7*, and *At-HpDGAT1-9*) were used for seed and oil analyses. The stable integration of pCAMBIA1303-*HpDGAT1* in *A. thaliana* genome and the transcription expression of *HpDGAT1* were checked by genomic PCR and qRT-PCR, respectively.

### Molecular Docking

AutoDock tool is a powerful method for identifying potential binding sites between 3D structure and ligand (Forli et al., 2016). The symmetrical half of the AST molecule (C20) was selected in docking process due to the main reasons that (1) the oversized C40 structure (compare to C16-C22 fatty acids); (2) AST esterification occurred on the hydroxyl group of the six-membered rings at both ends. In addition, FAs (C16:1 and C18:1n9) were used for AutoDock analysis as positive control because DGAT1 enzyme should have these FA-binding sites (Wang et al., 2020).

### Chemical Analysis

The HPLC method was applied to quantify the contents of different AST forms in *H. pluvialis* using the standard curve of AST (purchased from Sigma-Aldrich) at known concentrations (Cui et al., 2013). Total lipids extraction, TAG separation, and FA analysis from microalgae and higher plants were performed according to our previously described procedures (Liu et al., 2019; Cui et al., 2021). The Nile red staining was used to visualize the intracellular lipid bodies as an indicator of TAG formation according to previously described procedures (Siloto et al., 2009; Cui et al., 2021).

### Statistical Analysis

All experiments were biologically repeated three times to ensure reproducibility. The data were obtained as the mean value ± SD. Statistical analyses were performed using the SPSS statistical package (SPSS Inc., Chicago, IL, United States). Significant differences between treatments were statistically analyzed by paired-samples *t*-test. Statistical significance was achieved when *p* < 0.01.

## Results

### Cloning, Identification, and Sequence Analysis of *HpDGAT1* Gene

Based on the predicted partial coding sequence of *DGAT1* homolog (Supplementary Figure S1) from *H. pluvialis* transcriptome, a full-length cDNA fragment of *HpDGAT1* (GenBank no. MT612720) was cloned. The 1,811bp *HpDGAT1* in length contained a 1,560bp open reading frame (ORF) encoding 519 amino acids with MW of 58.7 kDa and pI of 9.8 (Supplementary Figure S2A). This protein was detected to have several conserved domains, such as chloroplast transduction peptide (CTP, 56 aa), TM helices (9 TMs), and phosphorylation sites (18) (Supplementary Figures S2B,E). All these analyses suggested that HpDGAT1 might be a membrane-bound DGAT1 enzyme localized in the chloroplast.

Sequence alignment of HpDGAT1 with other functional-known DGAT1s revealed 10 conserved motifs (CMs) in these proteins (Supplementary Figure S3). Four CMs were detected in the N-terminal of amino acids, including CM1 (the acyl-CoA binding), CM2 (putative thiolase acyl-enzyme intermediate signature), CM3, and CM4. Furthermore, a leucine zipper repeat motif with signature residues was also present in N-terminal of amino acids. However, HpDGAT1 lacked the N-terminal basic motif RRR present in higher plants or motif KRS substituted in other algae tested (Guihéneuf et al., 2011). The N-linked glycosylation sites (N-X-S/T) existed in DGAT1s from *Tropaeolum majus*, *Vernicia fordii*, and *Nicotiana tabacum* were absent in HpDGAT1, AtDGAT1, and BnDGAT1 (Caldo et al., 2017). The other six CMs (CM5-10) were discovered in the C-terminal of these DGAT1s, including CM6 involved in FA binding with a conserved tyrosine phosphorylation site, CM7 responsible for DAG-binding, and CM10, a putative C-terminal ER retrieval motif. In addition, CM5, CM8, and CM9 were found to locate near the active sites. All the above results indicate that HpDGAT1 is a member of DGAT1 family. The phylogenetic tree was constructed, which contained membrane-bound DGAT1s and DGAT2s, cytosolic DGAT3s, and dual-function WS/DGATs from algae and higher plants. HpDGAT1 was clustered into the DGAT1 family at bootstrap value of 100%, evidencing that the cloned cDNA encodes a typical DGAT1 protein (Supplementary Figure S4).

### Dynamic of *HpDGAT1* Expression, AST and TAG Accumulation in *H. pluvialis*

**Figure 1:**
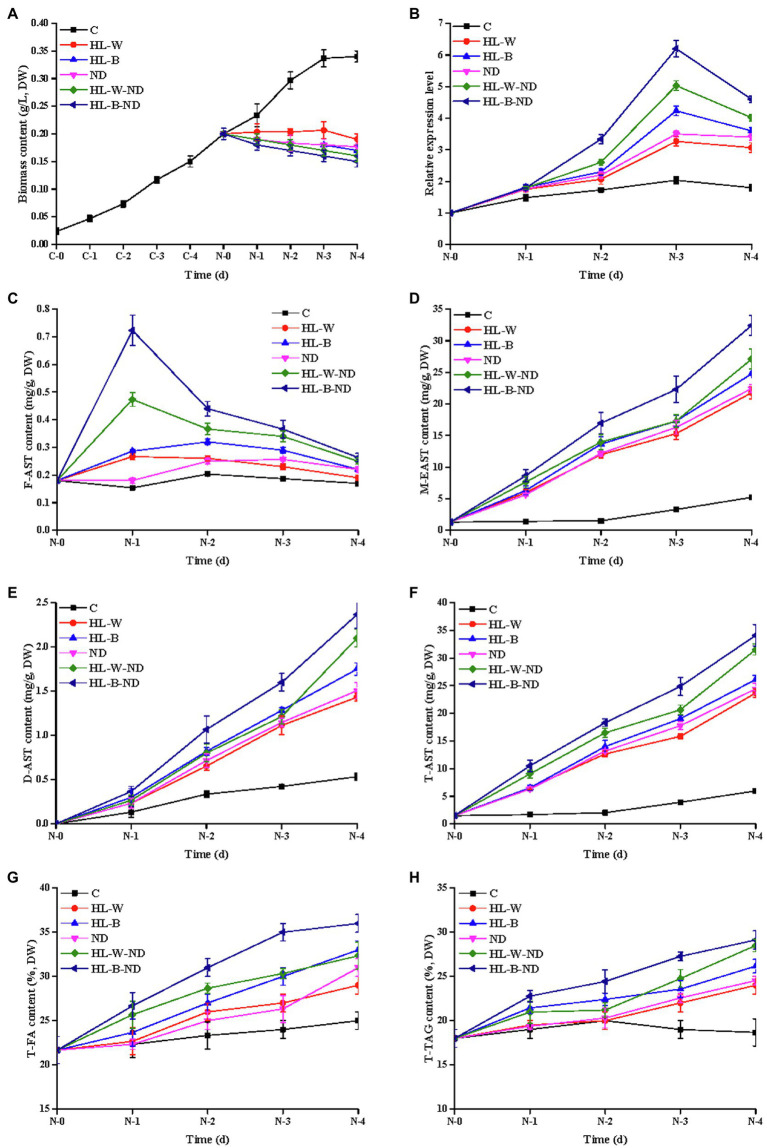
Analysis of algal growth, transcription expression level, AST, FA, and TAG contents under HL-W, HL-B, ND, HL-W-ND, and HL-B-ND conditions after 1, 2, 3, and 4 days. **(A)** Algal growth of *H. pluvialis.* **(B)** The transcriptional expression levels of *HpDGAT1*. **(C)** F-AST content. **(D)** M-EAST content. **(E)** D-EAST content. **(F)** T-AST content. **(G)** T-FA content. **(H)** T-TAG content. Data are means ± SE for three biological replicates.

To further understand the possible role of HpDGAT1 in AST and TAG accumulation, several physiological-biochemical assays were employed to examine the transcriptional expression of *HpDGAT1*, and the accumulation patterns of total AST (T-AST), total TAG (T-TAG), and total FA (T-FA) in *H. pluvialis* under HL-B, HL-W, ND, HL-B-ND, and HL-W-ND stresses (Figure 1).

As shown in Figure 1A, the HL-B, HL-W, ND, HL-B-ND, and HL-W-ND stresses intensively inhibited the algal growth in different degrees. Free AST (F-AST) content increased on 1 d under HL-B-ND, HL-W-ND, HL-B, and HL-W conditions and then decreased along with the induction time (2–4 d; Figure 1C). M-EAST content continuously increased along with the induction time (1–4 d), with the peak levels of 33.8, 27.6.22.3, 23.8, and 22.3 mg g^−1^ under stresses of HL-B-ND, HL-W-ND, ND, HL-B, and HL-W, respectively, on the final day (Figure 1D). This dynamic trend was also observed for D-EAST content despite the highest value was less than 3.0 mg g^−1^ (Figure 1E). T-AST content had the similar trend as M-EAST because M-EAST was the main form of ASTs (Figure 1F). Total TAG (T-TAG) content slowly increased from day 1 to 4 and then reached its maximum value of 29.5, 28.7, 25.2, 26.8, and 24.8%, respectively, for HL-B-ND, HL-W-ND, HL-B, HL-W, and ND treatments on 4 d, which were 159.5, 155.1, 136.2, 144.9, and 134.1% higher than that in the control (Figure 1H). The trend of change in T-FA content was highly similar with Total TAG accumulation (Figure 1G). The simultaneous accumulation of TAG and AST under HL-B, HL-W, ND, HL-B-ND, and HL-W-ND stresses encourages us to further explore the correlation between them and to identify the enzyme responsible for both EAST and TAG biosynthesis in *H. pluvialis*. Upon exposure of *H. pluvialis* cells to HL-B-ND, HL-W-ND, ND, HL-B, and HL-W stresses, the *HpDGAT1* mRNAs rapidly increased up to their maximum levels at 3 d exposure, with 3.1-, 2.5-, 1.7-, 2.1-, and 1.6-fold higher than that in the control, respectively (Figure 1B). The upregulation of *HpDGAT1* expression was positively concomitant with the increase of T-AST, T-TAG, and T-FA contents under the tested stress conditions, indicating that HpDGAT1 might play an important role in both TAG and AST accumulation in *H. pluvialis*, particularly under stress conditions.

### HpDGAT1 Can Restore TAG Biosynthesis in the Quadruple Mutant Yeast Strain H1246

**Figure 2:**
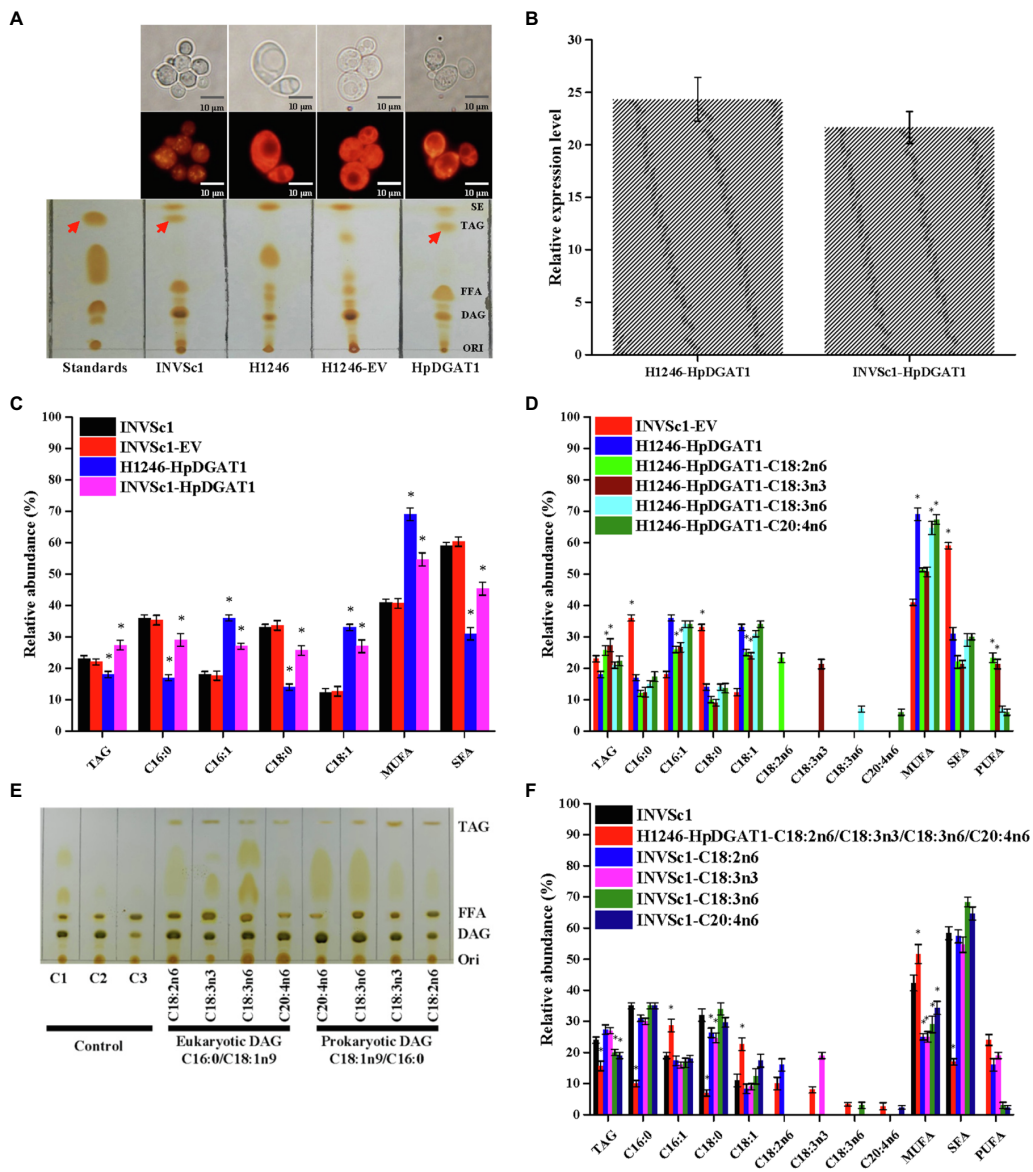
Functional characterization of *HpDGAT1* in *Saccharomyces cerevisiae* cells. **(A)** TLC and Nile red staining analysis of total lipids extracted from control *S. cerevisiae* (INVSc1), TAG-deficient *S. cerevisiae* (H1246), and H1246 cells transformed with *HpDGAT1* and Empty vector (EV). **(B)** The transcriptional expression levels of *HpDGAT1* in H1246 and INVSc1 cells transformed with *HpDGAT1*. The gene expression levels were normalized to the endogenous *ScACTIN* gene. **(C)** TAG contents and relative abundance of fatty acids in INVSc1 and H1246 cells transformed with *HpDGAT1*. **(D)** Relative abundance of fatty acids in H1246 cells transformed with *HpDGAT1* by the feeding of free fatty acids of C18:2n6, C18:3n3, C18:3n6, and C20:4n6, respectively, after a 24 h cultivation. **(E)** The *in vitro* substrate specificities of HpDGAT1 for acyl CoAs. C1, microsome plus eukaryotic DAG; C2, microsome plus prokaryotic DAG; and C3, microsome. **(F)** Relative abundance of fatty acids in INVSc1 cells by the feeding of free fatty acids of C18:2n6, C18:3n3, C18:3n6, and C20:4n6, respectively, after 24 h cultivation.

To identify the HpDGAT1 enzymatic activity, the *HpDGAT1* coding sequence was heterologously overexpressed in the TAG-deficient yeast quadruple mutant strain H1246. The wild-type yeast strain INVSc1 and the mutant H1246 strain carrying the empty vector (H1246-EV) were used as positive and negative controls, respectively. A qRT-PCR analysis displayed that *HpDGAT1* gene was effectively expressed in the transgenic yeast cells. TLC separation of total lipids (Figure 2A) and Nile Red staining (Figure 2B) of oil bodies both revealed that TAG were largely accumulated in the yeast cells overexpressing *HpDGAT1*. TAG content and FA composition were similar in both wild-type INVSc1 and INVSc1-EV cells (Figure 2C). Notably, compared to wild-type INVSc1, higher levels of TAG (26.6%) were obtained in the INVSc1 cells overexpressing *HpDGAT1* due to the contribution of both endogenous yeast DGAT and exogenous algal HpDGAT1. However, TAG content (18.8%) in the *HpDGAT1-*transformed H1246 was lower than that in INVSc1 and INVSc1-EV (21.9%) although HpDGAT1 restored TAG synthesis in the transgenic H1246 cells. The possible reason may be that acyl-CoA substrates in yeast cells are not suitable for HpDGAT1. Fatty acid profiles in TAGs detected by GC showed that levels of monounsaturated fatty acyl-CoAs (MUFAs, C16:1 and C18:1) were much higher in both the transgenic H1246 and INVSc1 cells overexpressing *HpDGAT1* than that in the wild-type INVSc1 and INVSc1- EV cells, indicating that HpDGAT1 may have higher substrate preference for MUFAs (C16:1 and C18:1) than saturated fatty acids (SFAs, C16:0 and C18:0) in yeast cells.

### HpDGAT1 Preferred Substrates of PUFAs for TAG Assembly in Yeast Assays With Feeding of Exogenous Fatty Acids

Normally, the host yeast cells just synthesized four major fatty acids including C16:0, C18:0, C16:1, and C18:1, but not the PUFAs, such as C18:2n6, C18:3n3, C18:3n6, and C20:4n6 enriched in *H. pluvialis* (Chen et al., 2015; Ma et al., 2018). In order to further identify substrate preference to those PUFAs synthesized in *H. pluvialis*, the *HpDGAT1-*transgenic yeast H1246 cells were cultured in the medium supplemented with each of those PUFAs, respectively. Interestingly, compared to yeast cells without feeding of any exogenous fatty acids, the *HpDGAT1-*transgenic yeast H1246 cells with PUFAs supplement accumulated high levels of TAGs with different degrees of enhancement for various PUFAs feeding. The increased level of TAGs was ordered as C18:3n3>C18:2n6>C20:4n6>C18:3n6 (Figure 2D). The above results indicated that HpDGAT1 could import these free PUFAs for sequestration into TAG. Additionally, PUFAs supplement also adjusted the FA profiles in TAGs. Much higher levels of PUFAs (C18:3n3 and C18:2n6) were incorporated into TAGs in the *HpDGAT1*-transgenic H1246 cells when fed with C18:3n3 or C18:2n6, respectively (Figure 2D), which were at the cost of lower levels of MUFAs (C16:1 and C18:1) and SFAs (C16:0 and C18:0). Conversely, the FA profiles in the *HpDGAT1*-transgenic H1246 cells were similar between exogenous C20:4n6 or C18:3n6 fed, respectively, and non-fed treatments (Figure 2D). Overall, these FA profiles demonstrated that HpDGAT1 had a stronger substrate preference for C18:3n3 and C18:2n6 than C20:4n6 and C18:3n6.

### *In vitro* Acyl-CoA Substrate Specificity of HpDGAT1

Alternatively, the import capacities for various PUFAs in yeast might be responsible for the different FA profiles. To test this possibility, two strategies were applied. Firstly, the yeast microsome was prepared and *in vitro* acyl-CoA substrate specificity for HpDGAT1 was analyzed. The prokaryotic DGA (C18:1n9/C16:0) and eukaryotic DAG (C16:0/C18:1n9) were used as an acyl acceptor for the *in vitro* assay. The PUFAs including C18:2n6, C18:3n3, C18:3n6, and C20:4n6 were examined. As shown in Figure 2E, C18:2n6 and C18:3n3 but not C18:3n6 and C20:4n6 were the preferred substrates for HpDGAT1 indicated by the obvious TAG spots. HpDGAT1 had the greatest activity toward C18:3n3, followed by C18:2n6, C18:3n6, and C20:4n6 when eukaryotic DAG (C16:0/C18:1n9) was used. The similar trend was also presented when prokaryotic DGA (C18:1n9/C16:0) was used. Again, these above results implied that HpDGAT1 had a stronger substrate preference for C18:3n3 and C18:2n6 than C20:4n6 and C18:3n6 *in vitro*. Secondly, the wild-type INVSc1 yeast cells were cultured in the medium supplemented with each of those PUFAs, respectively (Figure 2F). Higher levels of TAG were obtained in the INVSc1 cells when fed with C18:2n6 and C18:3n3 than no fed treatment. Meanwhile, the FA profiles in the INVSc1 cells were changed. Briefly, the contents of C18:3n3 and C18:2n6 were increased at the expensive of MUFAs (C16:1 and C18:1) while the SFAs (C16:0 and C18:0) contents remained constant. However, the lower levels of TAG were observed in the INVSc1 cells when fed with C18:3n6 and C20:4n6 than no fed treatment. The FA profiles in these feeding INVSc1 cells remained unchanged. It is important to note as well that the TAG accumulation was obviously restricted when the HpDGAT1-transgenic yeast H1246 cells were cultured in the medium supplemented with a mixture of four PUFAs. Overall, these FA profiles demonstrated that HpDGAT enzyme activity and import capacities for various PUFAs together determined the acyl-CoA substrate specificity for HpDGAT1 in yeast cells.

### Overexpression of *HpDGAT1* in Microalga *C. reinhardtii* Significantly Enhances TAG Accumulation

**Figure 3:**
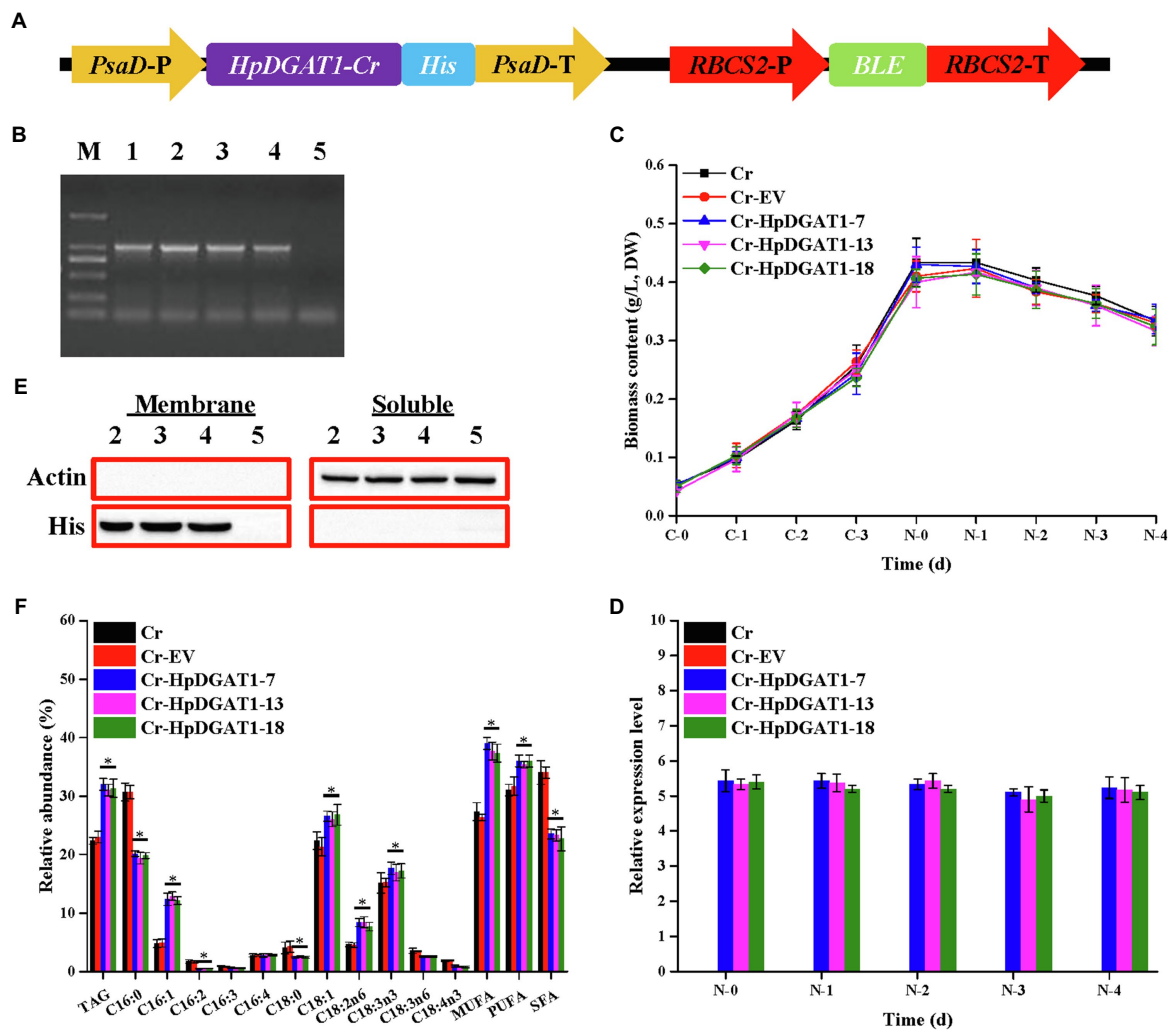
Genetic engineering of *HpDGAT*1 in *Chlamydomonas reinhardtii* cells. **(A)** Construct of the expression vector. *PsaD*-P, promoter of *PsaD* gene; His tag, 6-His encoding gene; *PsaD*-T, terminator of *PsaD* gene; *RBCS2*-P, promoter of *RBCS2* gene; *Ble*, the *bleomycin*-resistant gene; and *RBCS2*-T, terminator of *RBCS2* gene. **(B)** Genomic level of *HpDGAT1* in *C. reinhardtii* cells. 1: pDB124-*HpDGAT1*; 2: Cr + *HpDGAT1*-7; 3: Cr + *HpDGAT1*-13; 4: Cr + *HpDGAT1*-18; and 5: Cr + EV. **(C)** Time course of biomass content under control and ND conditions. **(D)** The transcriptional expression levels of *HpDGAT1* in *C. reinhardtii* cells. The gene expression levels were normalized to the endogenous *CrACTIN* gene. **(E)** Western blotting. Soluble and membrane proteins were separated and used for blotting. 2: Cr + *HpDGAT1*-7; 3: Cr + *HpDGAT1*-13; 4: Cr + *HpDGAT1*-18; and 5: Cr + EV. **(F)** TAG content and relative abundance of fatty acids in *C. reinhardtii* cells transformed with *HpDGAT1.*

To explore engineering potential of *HpDGAT1* to modulate TAG biosynthesis in microalgae, the *HpDGAT1* gene with codon preference optimized (Supplementary Figure S5) was transformed into model algal *C. reinhardtii* CC849 using the nuclear transformation expression vector pDB124 under the control of the endogenous promoter and terminator (Figure 3A; Supplementary Figure S6). A number of transgenic *C. reinhardtii* lines were successfully obtained. After screening over 20 putative algal transformants and identification by genomic PCR, three *HpDGAT1*-transgenic algal lines (*Cr-HpDGAT1-7*, *Cr-HpDGAT1-13*, and *Cr-HpDGAT1-18*) were selected for further analysis (Figure 3B). The qRT-PCR analysis showed that heterologous *HpDGAT1* gene was effectively expressed in these three transgenic algal lines (Figures 3C,D). Under ND conditions, the expression level of *HpDGAT1* was approximately 5.5-fold higher in these three algal lines than that of the control, without detectably affecting algal growth (Figures 3C,D). In addition, the expression of HpDGAT1 protein was examined using His-tagged antibodies *via* Western blot analysis. As shown in Figure 3E, the target bands were present in the membrane protein fractions prepared from the three transgenic algal lines, but absent in the soluble protein samples, evidencing that HpDGAT1 is a trans-membrane enzyme (Figure 3E; Supplementary Figure S7).

It is not surprising that the TAG contents in the transgenic algal lines overexpressing *HpDGAT1* were significantly increased by ~1.5-fold under ND conditions (Figure 3F), which may be partially due to the increased enzyme protein resulted from high transcription expression of *HpDGAT1* (Figure 3D). In addition, the present data also indicated that the heterologous overexpression of *HpDGAT1* also affected the FA profiles in TAGs (Figure 3F). The accumulation of MUFAs (C16:1 and C18:1) and PUFAs (C18:2n6 and C18:3n3) was significantly increased in the *HpDGAT1-*transgenic algal lines, accompanied by reduction of SFAs (C16:0 and C18:0) and PUFAs (C16:2, C18:3n6, and C18:4n3). There was no significant change in C16:4 and C16:3 contents between the transgenic algal lines and the controls. These results again indicated that HpDGAT1 had a stronger preference for MUFAs (C16:1 and C18:1) and PUFAs (C18:2n6 and C18:3n3) over SFAs (C16:0 and C18:0) and PUFAs (C16:2, C18:3n6, and C18:4n3), showing the application of HpDGAT1 in algal lipid engineering.

### Overexpression of *HpDGAT1* in Higher Plant *A. thaliana* Greatly Promotes TAG Accumulation

**Figure 4:**
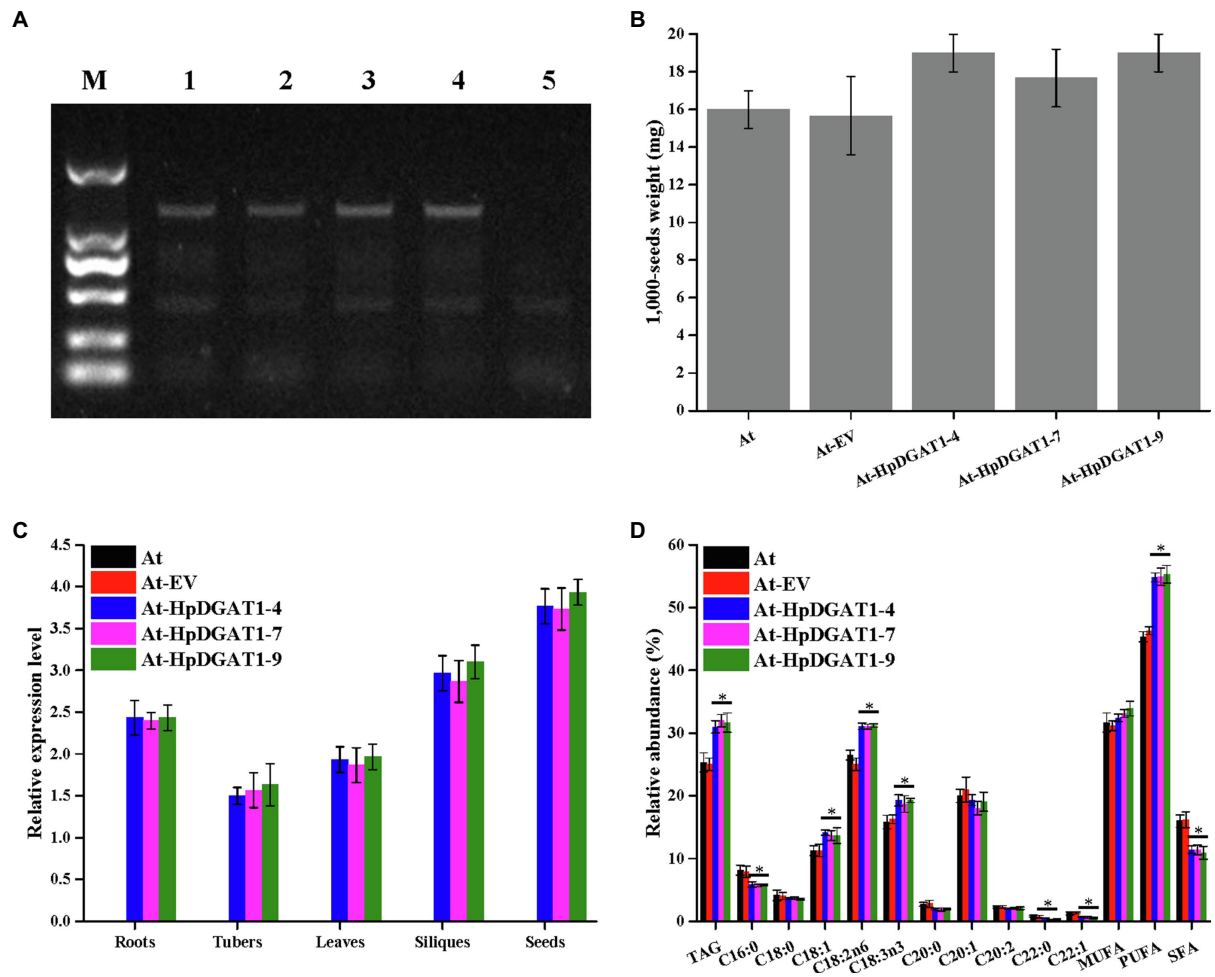
Genetic engineering of *HpDGAT*1 in *Arabidopsis thaliana*. **(A)** Genomic detection of *HpDGAT1* in *A. thaliana*. 1: pCAMBIA1303-*HpDGAT1*; 2: At-*HpDGAT1*-4; 3: At-*HpDGAT1*-7; and 4: At-*HpDGAT1*-9; 5: At-EV. **(B)** Average 1,000-seed weight (expressed as milligrams of weight/1,000 seeds) of transgenic *Arabidopsis* T2 seeds. **(C)** The transcriptional expression levels of *HpDGAT1* in *A. thaliana*. The gene expression levels were normalized to the endogenous *AtACTIN* gene. **(D)** TAG content and relative abundance of fatty acids in *A. thaliana* transformed with *HpDGAT1.*

To explore engineering potential of *HpDGAT1* to improve TAG yield and FA composition in higher plants, *HpDGAT1* gene was heterologously expressed in *A. thaliana*. Three T2 transgenic lines (*At-HpDGAT1-4*, *At-HpDGAT1-7*, and *At-HpDGAT1-9*) were selected for further analysis. The 1,000-seed weight was largely enhanced compared to the controls (Figures 4A,B). This increase may be resulted from high activities of HpDGAT1 which pulled large amount of carbon flux into TAG biosynthesis in seed. The qRT-PCR results indicated that the *HpDGAT1* transcripts were highly accumulated in the tested organs of the transgenic lines despite of different expression patterns (Figure 4C). Accordingly, the total TAG contents in seeds were increased from 25.23% in the control plants up to 30.13–32.64% in the *HpDGAT1-*overexpressed *Arabidopsis* lines (Figure 4D). Moreover, the heterologous overexpression of *HpDGAT1* gene also resulted in alternation of FA profiles in TAGs in seeds of the transgenic *Arabidopsis* (Figure 4D), just like the cases in the yeast assay with feeding of exogenous FAs and *HpDGAT1*-overexpressed *C. reinhardtii* lines, respectively, described above (Figures 2D, 3F). Briefly, the abundances of C18:1, C18:2n6, and C18:3n3 were dramatically increased in the transgenic *Arabidopsis* seeds while C20:1, C20:2, and C22:1 maintained stable in both the transgenic lines and the control plants. For SFAs, C16:0 and C22:0 content decreased in the transgenic lines while C18:0 and C20:0 levels were not changed between the transgenic and control plants. These results revealed that the *HpDGAT1-*overexpression can increase both seeds weight and TAG enrichment in *Arabidopsis*, once again indicating that HpDGAT1 has a high substrate specificity for C18:1, C18:2n6, and C18:3n3. In summary, our data evidence that *HpDGAT1* gene can be served as the target in genetic engineering to effectively improve oil yield and fatty acid composition in oilseeds or other higher plants.

### Molecular Docking Reveals the Binding Sites Present for HpDGAT1 Enzyme and AST Structure

**Figure 5:**
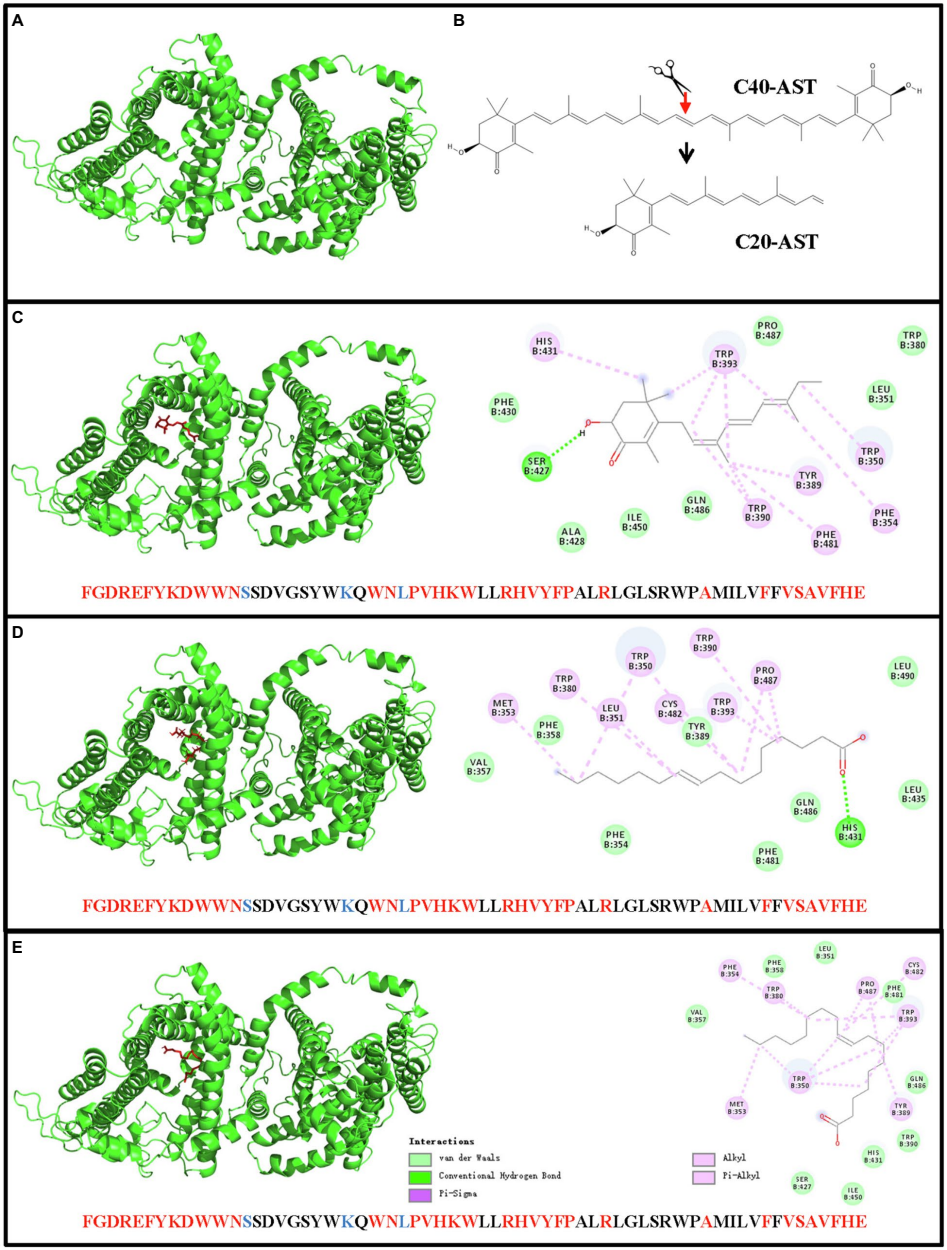
Docking of AST molecular and 3D model of HpDGAT1 using AutoDock software. **(A)** The 3D model of HpDGAT1. **(B)** AST molecular. **(C)** Binding sites between AST and HpDGAT1. **(D)** Binding sites between C16:1 and HpDGAT1. **(E)** Binding sites between C18:1 and HpDGAT1. The predicted AST-binding sites were located in CM6, CM7, and CM8. The conserved amino acid residues and substitutions were indicated by red and blue color, respectively.

Molecular docking (AutoDock analysis) was performed to explore the binding sites between AST structure and 3D structure of HpDGAT1. SWISS-MODEL server was employed to successfully generate the 3D structure for HpDGAT1 using crystal structure of human DGAT1 in complex with an oleoyl-CoA substrate as the template (Figure 5A; Wang et al., 2020). The symmetrical half of the AST molecule (C20, Figure 5B) got docked into the predicted 3D model of HpDGAT1 (Figure 5C) by van der waals force, conventional hydrogen bond, alkyl, Pi-alkyl, and Pi-sigma. The predicted binding amino acids sites were listed in Figure 5C, which were located in CM6-8 (Supplementary Figure S3). Interestingly, there were some organism-specific amino acid sequences and amino acid substitutions in the above CMs, which might play important role in AST esterification for HpDGAT1. In order to test the reliability of the AutoDock analysis, some other binding sites between fatty acyl-CoA (C16:1 and C18:1) and 3D structure of HpDGAT1 were also predicted. These binding sites should be existed in the DGAT1 enzyme (Wang et al., 2020). As expected, some fatty acyl-CoA-binding sites were predicted, which were listed in Figures 5D,E. These predicted sites were highly consistent with the reported CoA-binding signature in human DGAT1 (Wang et al., 2020). All these results provide another key clue for potentially functional role of HpDGAT1 in AST esterification despite further biological experiments, e.g., the expression of *HpDGAT1* in AST-producing yeast, algal, and bacteria strains, are necessary in future to support this hypothesis.

## Discussion

The unicellular green alga *H. pluvialis* is well characterized for its ability to produce high levels of natural EASTs and TAGs in a tightly associated model under stress conditions, representing an ideal industrial producer of both oils and ASTs (Lorenz and Cysewski, 2000; Chen et al., 2015; Bilbao et al., 2020) despite the detail mechanism underlying TAG and AST biosynthesis remains unclear in this alga. Based on the finding that the predominant ASTs are deposited as the esterified forms (M-EAST and D-EAST) in TAG-filled OBs in algal cells, it has been hypothesized that TAG biosynthetic enzymes may be the candidates for AST esterification in AST-producing algae *H. pluvialis* and *C. zofingiensis* (Chen et al., 2015; Ma et al., 2018; Zhao et al., 2020). To date, no such enzyme was identified. We reported here that a novel HpDGAT1 was systematically characterized by *in vivo* and *in silico* assays on its function in TAG biosynthesis and possible role in AST esterification.

Intriguingly, the results revealed that HpDGAT1 was a membrane-bound DGAT1 enzyme localized in the chloroplast of *H. pluvialis* (Supplementary Figures S2B,E), which is consistent with previous reports that DGAT1s had six to nine TMs (Xu et al., 2018) and NoDGAT1A located in chloroplast endoplasmic reticulum (cER) in *Nannochloropsis oceanica* (Wei et al., 2017). In addition, total of 18 phosphorylation sites were predicted in HpDGAT1 protein, demonstrating that phosphorylation might play important roles in HpDGAT1 enzyme activity like the cases in previously characterized mouse DGAT1 (Yen et al., 2008), TmDGAT1 (Xu et al., 2008), and BnDGAT1 (Caldo et al., 2018). Despite of a low sequence identity at the N-terminal of amino acids, all DGAT1s share a common catalytic center containing acyl-CoA-binding motif, DAG-binding motif, the FA-binding motif, and a putative C-terminal ER retrieval motif (Guihéneuf et al., 2011). It is worth noting that the variable region of N-terminus is the typical characteristic of DGAT1s. Such variable region might determine distinct functions of DGAT1s in different organisms (Guihéneuf et al., 2011). Sequence alignment and phylogenetic analysis with HpDGAT1 and other functional-known DGATs again revealed that HpDGAT1 is a member of DGAT1 family (Supplementary Figures S3, S4).

Previous studies showed that M-AST was the predominant AST form (Miao et al., 2006; Yang et al., 2021) in *H. pluvialis.* Moreover, HL and ND stresses effectively induced accumulation of AST and TAG in this alga (Chen et al., 2015; Ma et al., 2018; Zhang et al., 2018; Zhao et al., 2020; Cui et al., 2021). Similarly, our data demonstrated that the content of T-AST continuously increased along with the induction time under HL-B-ND, HL-W-ND, HL-B, HL-W, and ND treatments (Figures 1C–F). In addition, T-TAG and T-FA contents in algal cells under those stresses slowly increased from day 1 to 4 and then reached its maximum value on 4 d (Figures 1G,H). Collectively, our data again evidence that that TAG and AST are simultaneously accumulated in *H. pluvialis* under the stress conditions.

In view of a speculation that DGAT enzyme may be responsible for both AST esterification and TAG biosynthesis in *H. pluvialis* (Chen et al., 2015; Ma et al., 2018; Zhao et al., 2020), we further examined *HpDGAT1* expression profiles in *H. pluvialis* under various stress conditions. As shown in Figure 1B, *HpDGAT1* mRNAs rapidly increased up to their maximum levels at 3 d exposure with different patterns under these stresses. This expression pattern of *HpDGAT1* gene is supported by a previous report that HL-W was more effective than HL-B in inducing the expression of a putative *DGAT1* in *H. pluvialis* (Ma et al., 2018). A number of studies showed that expressions of TAG biosynthesis enzyme genes were induced by various stresses. For example, *CeDGAT1* transcript was increased in *C. ellipsoidea* under ND stress (Guo et al., 2017). Moreover, the expressions of *CrDGAT1*, *CrDGTT1*, and *CrPDAT1* genes were upregulated by ND stress in *C. reinhardtii* (Liu et al., 2016). Analogously, some *DGAT1* genes were induced to express in *N. oceanica* and *C. zofingiensis* under ND and HL stress conditions (Li et al., 2014; Wei et al., 2017; Ma et al., 2018; Mao et al., 2019). In our study, the upregulation of *HpDGAT1* expression was positively concomitant with the increase of T-AST, T-TAG, and T-FA contents under the tested stress conditions, indicating that HpDGAT1 might play an important role in both TAG and AST accumulation in *H. pluvialis*, particularly under stress conditions.

An essay using TAG-deficient quadruple mutant yeast strain H1246 is a powerful tool to identify the DGAT enzymatic activity (Guihéneuf et al., 2011). As shown in Figure 2, *HpDGAT1* gene was effectively expressed in the transgenic H1246 yeast cells, and such transgenic yeast cells accumulated lots of TAGs. Intriguingly, compared to the wild-type INVSc1, higher levels of TAG (26.6%) were obtained in the INVSc1 cells overexpressing *HpDGAT1* due to the contribution of both endogenous yeast DGAT and exogenous HpDGAT1. However, TAG content (18.8%) in the *HpDGAT1-*transformed H1246 was lower than that in INVSc1 and INVSc1-EV (21.9%), which might be explained by the inappropriate acyl-CoA substrates in yeast cells. The similar phenomenon has been observed in previous studies (Liu et al., 2016; Mao et al., 2019). Further fatty acid profiles analysis showed that HpDGAT1 may have substrate preference for MUFAs (C16:1 and C18:1) in yeast cells (Figure 2C).

Generally, the diversity of substrate preference of DGATs for acyl-CoA depends not only on the enzyme features, but also on the FAs available in the species tested (Wei et al., 2017; Mao et al., 2019). Yeast also provides a good system to test DGAT substrate preference due to it can absorb exogenous fatty acids from the medium and then convert the FAs to their respective acyl-CoA derivatives, which consequently are incorporated into TAGs by DGAT in yeast cells. The PUFAs (C18:2n6, C18:3n3, C18:3n6, and C20:4n6) were enriched in *H. pluvialis* but absent in the yeast (Chen et al., 2015; Ma et al., 2018). These PUFAs can be added into yeast medium, respectively, to identify the DGAT substrate specificity. Overall, FA profiles detected in the yeast cells fed by each of these exogenous FAs demonstrated that HpDGAT1 had a stronger substrate preference for PUFAs (C18:3n3 and C18:2n6) and MUFAs (C16:1 and C18:1). Considering that C18:2n6 and C18:3n3 were highly accumulated in *H. pluvialis* (Chen et al., 2015; Ma et al., 2018), it is reasonable to speculate that the HpDGAT1 may have a great potential in gene engineering to specifically increase C18:2n6- and C18:3n3-enriched TAG production in other oleaginous microalgae.

Alternatively, the import capacities for various PUFAs in yeast might be responsible for the above different FA profiles. Two strategies were applied to test this possibility. Firstly, the *in vitro* acyl-CoA substrate specificity assay for HpDGAT1 indicated that C18:2n6 and C18:3n3 but not C18:3n6 and C20:4n6 were the preferred substrates for HpDGAT1, which was insistent with feeding test in H1246 cells with HpDGAT1 overexpression (Figure 2E). Secondly, the results from feeding test for wild-type INVSc1 yeast cells implied that higher levels of TAG were obtained in the INVSc1 cells when fed with C18:2n6 and C18:3n3 but the lower levels of TAG were observed in the INVSc1 cells when fed with C18:3n6 and C20:4n6 (Figure 2F). Therefore, these FA profiles demonstrated that HpDGAT enzyme activity and import capacities for various PUFAs together determined the acyl-CoA substrate specificity for HpDGAT1 in yeast cells.

To further evaluate the engineering potential of HpDGAT1 in modulating TAG biosynthesis, we employed *C. reinhardtii*, a unicellular eukaryotic model species, for transformation of *HpDGAT*1 gene since this species is the ideal host for functional identification of any foreign DGAT due to all physiological processes taking place within a cell (Liu et al., 2016; Mao et al., 2019). As shown in Figures 3A–E, the three transgenic *C. reinhardtii* lines were successfully obtained. The heterologous *HpDGAT1* gene was effectively expressed at both transcription and protein levels in these algal lines without significant difference in growth performance compared to the wild-type control. In addition, HpDGAT1 proteins were present in the membrane protein fractions, but absent in the soluble protein samples (Figure 3E; Supplementary Figure S7), evidencing that HpDGAT1 is a trans-membrane enzyme. More importantly, TAG contents in the transgenic algal lines overexpressing *HpDGAT1* were significantly increased by ~1.5-fold under ND conditions (Figure 3F), indicating that HpDGAT1 can highly function in the ectopic host (Figure 3D). The heterologous overexpression of *HpDGAT1* also affected the FA profiles in TAGs in the *C. reinhardtii* cells (Figure 3F). The accumulation of MUFAs (C16:1 and C18:1) and PUFAs (C18:2n6 and C18:3n3) was significantly increased in the *HpDGAT1-*transgenic algal lines, accompanied by reduction of SFAs (C16:0 and C18:0) and PUFAs (C16:2, C18:3n6, and C18:4n3). The present data again indicated that HpDGAT1 had a stronger preference for MUFAs (C16:1 and C18:1) and PUFAs (C18:2n6 and C18:3n3). This strong substrate preference of HpDGAT1 was not fully examined for NoDGAT1A and CzDGAT1A when they were overexpressed, respectively, in *C. reinhardtii* UVM4 (Wei et al., 2017) and *N. oceanica* (Mao et al., 2019) despite their expressions resulted in enhancement of TAG accumulation in the hosts.

Finally, we overexpressed *HpDGAT1* in *Arabidopsis thaliana* to investigate whether this gene can be also engineered into higher plants to improve TAG yield and FA composition in seeds. Our data displayed that TAG content increased greatly in *Arabidopsis* seeds (Figure 4D), which is consistent with higher expression of *HpDGAT1* gene in developing seeds (Figure 4C). Previously, *DGAT’s* expression levels were detected to positively correlate with oil deposition in developing seeds in higher plants (Li et al., 2010). Theoretically, high activities of DGAT1s could enlarge TAG pools, which consequently pull more carbon flux into TAG biosynthesis in seed. Moreover, *HpDGAT1-*overexpression led to significant alternation of FA profiles in *Arabidopsis* seeds by enhanced accumulation of MUFAs- and PUFAs-rich TAGs (Figure 4D). In agreement with our findings, some microalgal DGAT1s were previously reported to increase oil accumulation in the ectopic host when they were overexpressed, with *CeDGAT1* in *A. thaliana* and *B. napus* (Guo et al., 2017) and *NoDGAT1* in *Arabidopsis* seeds (Zienkiewicz et al., 2017). Similarly, overexpression of *EgDGAT2* from *Elaeis guineensis* in *A. thaliana* enhanced the contents of C18:2 and C18:3 in seed oils followed with the reduction of C18:0 and C20:0 levels in the transgenic seeds (Jin et al., 2017). The descriptions above evidence that *HpDGAT1* gene can be served as a better target in genetic engineering to effectively improve oil yield and fatty acid composition in higher oilseeds and

Although some studies suggested that DGATs may be the crucial enzymes involving in EAST biosynthesis in *H. pluvialis* (Chen et al., 2015; Ma et al., 2018; Zhao et al., 2020), no direct biochemical evidence has been obtained for this function yet. Recently, all 10 *CzDGATs* were expressed in the AST-producing yeast strain to investigate if these enzymes are responsible for EAST biosynthesis. However, no newly synthesized EAST was detected in the transgenic host, indicating the null function of CzDGATs in AST esterification (Mao et al., 2019). Considering that the difference in genetic traits and AST biosynthesis pathway between AST-producing algal strains *C. zofingiensis* and *H. pluvialis*, it was speculated that HpDGAT1 from *H. pluvialis* may function in AST esterification (Zhang et al., 2020). In this study, molecular docking (AutoDock analysis) was performed to explore the binding sites between AST structure and 3D structure of HpDGAT1. The results indicated that fatty acyl-CoA-binding sites existed in the HpDGAT1 enzyme, similarly in human DGAT1 (Wang et al., 2020). The AST-binding sites were also predicted in HpDGAT1 protein sequences (Figure 5). Moreover, structure–function analysis of the hydrophilic N-terminal domain of HpDGAT1 indicated that the N-terminal domain of HpDGAT1 may play important roles in maintaining enzyme performance, like the case of CzDGAT1 in *C. zofingiensis* (Xu et al., 2020). All these results provide a new clue for functional role of HpDGAT1 in AST esterification despite further biological experiments, such as the expression of *HpDGAT1* in AST-producing yeast, algal, and bacteria strains, are necessary to support this hypothesis.

**Figure 6:**
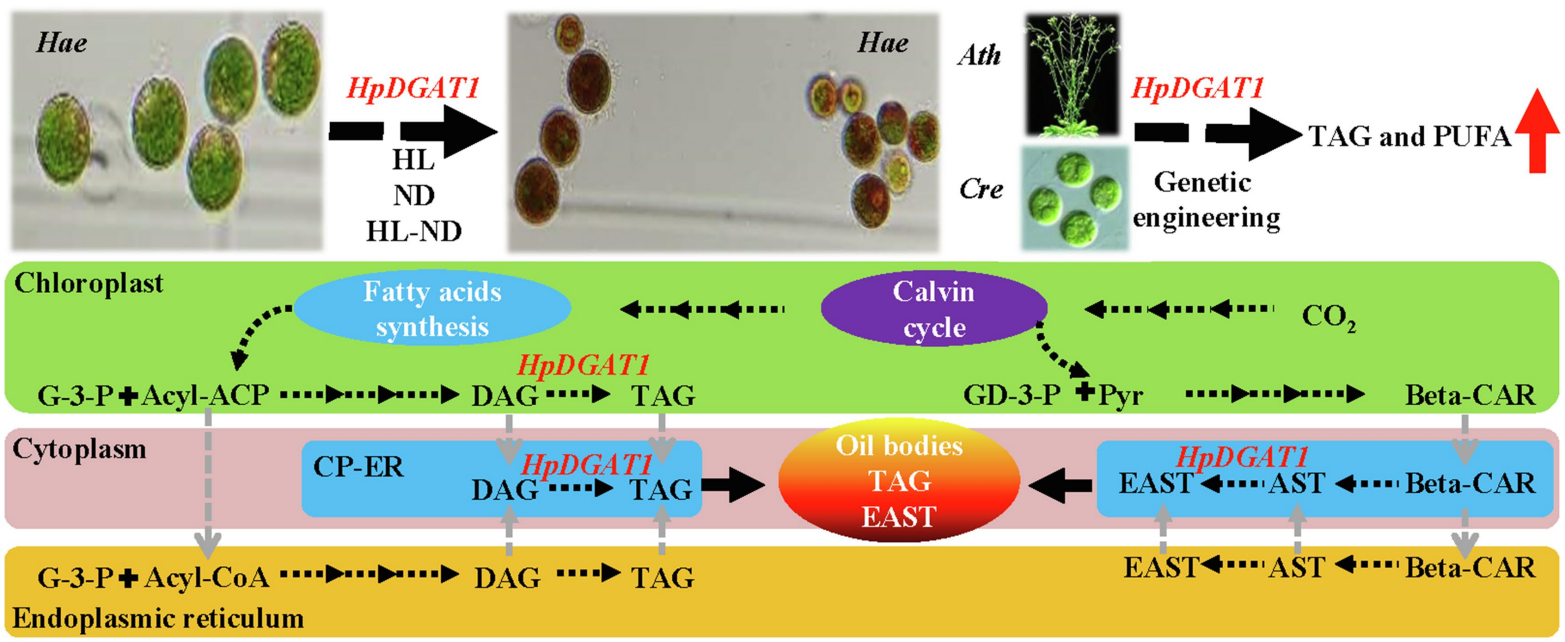
A hypothesized working model illustrating the potential dual-function of HpDGAT1 in TAG and EAST biosynthesis in *H. pluvialis.* Briefly, under stress conditions, the *HpDGAT1* transcript was obviously increased, which was accompanied by the significant increase of TAG and EAST contents. The genetic engineering of *HpDGAT1* in *Chlamydomonas reinhardtii* (*Cre*) and *Arabidopsis thaliana* (*Ath*) significantly enhanced the MUFAs- and PUFAs-rich TAG accumulation. The *de novo* synthesized fatty acyls constitute the acyl-ACP or acyl-CoA pools, which consequently enter Kennedy pathway for the synthesis of TAG in cytoplasm endoplasmic reticulum (ER) or chloroplast ER (cER). HpDGAT1 could catalyze the prokaryotic TAG accumulation in chloroplast or probably located in cER (cER is expressed as an elbow-like line linking ER and chloroplast envelope) and utilized both the prokaryotic and eukaryotic DAGs for TAG assembly. In addition, EASTs might be synthesized in chloroplast ER or cytoplasm ER by HpDGAT1 enzyme, which is dependent on availability of free AST and acyl-CoAs. Finally, TAG and EAST are together packed into oil bodies.

In summary, a potentially dual-function *HpDGAT1* gene was identified in *H. pluvialis*. The dynamic of *HpDGAT1* expression is highly associated with AST and TAG accumulations under HL-W, HL-B, ND, HL-B-ND, and HL-W-ND stress conditions, respectively. HpDGAT1 has a strong enzymatic activity catalyzing TAG synthesis, and also a higher substrate specificity for UFAs, particularly PUFAs (C18:2n6 and C18:3n3). The potential applications in oil engineering in microalgal cells and *Arabidopsis* seeds were determined, showing that it can be used as an ideal target gene for effectively improving oil yields and FA profiles. Interestingly, the binding sites between AST and HpDGAT1 protein structure were predicted, providing a strong evidence for HpDGAT1’s function in AST esterification in *H. pluvialis*. Taken together, a working model is proposed for the potentially dual-functions of HpDGAT1 in *H. pluvialis* (Figure 6). The *de novo* synthesized fatty acyls constitute the acyl-ACP or acyl-CoA pools, which consequently enter Kennedy pathway for the synthesis of TAG in cytoplasm endoplasmic reticulum (ER) or chloroplast ER (cER). Therefore, we speculated that HpDGAT1 could catalyze the prokaryotic TAG accumulation in chloroplast or probably located in cER (cER is expressed as an elbow-like line linking ER and chloroplast envelope) and utilized both the prokaryotic and eukaryotic DAGs for TAG assembly. In addition, EASTs might be synthesized in chloroplast ER or cytoplasm ER by HpDGAT1 enzyme, which is dependent on availability of free AST and acyl-CoAs. Finally, TAG and EAST are together packed into oil bodies. Future studies are necessary to improve and amend this working model. Our findings on HpDGAT1 provide new insights into deep understanding of TAG and EAST synthesis in *H. pluvialis*.

## Data Availability Statement

The datasets presented in this study can be found in online repositories. The names of the repository/repositories and accession number(s) can be found in the article/Supplementary Material.

## Author Contributions

XJ, SQ, and RL conceived the idea and revised the manuscript. HC, WX, XZ, CZ, and YC performed the experiments. JX, CJ, and CZ analyzed the data. HC and WX wrote the manuscript. All authors contributed to the article and approved the submitted version.

## Funding

This study was supported by the National Natural Science Foundation of China (31902394 and 41876188), the Key Research and Development Planning Project of Shanxi Province (201803D31063), the Applying Basic Research Planning Project of Shanxi Province (201801D221250), the Major Basic Research Program of Shandong Province Natural Science Foundation (ZR2018ZB0210), the Key Research and Development Planning Project of Jinzhong City (Y192012), and the Science and Technology Innovation Planning Project of Shanxi Agricultural University (2018YJ16).

## Conflict of Interest

The authors declare that the research was conducted in the absence of any commercial or financial relationships that could be construed as a potential conflict of interest.

## Publisher’s Note

All claims expressed in this article are solely those of the authors and do not necessarily represent those of their affiliated organizations, or those of the publisher, the editors and the reviewers. Any product that may be evaluated in this article, or claim that may be made by its manufacturer, is not guaranteed or endorsed by the publisher.

## Footnotes

^1^ http://www.larodan.com/

^2^ http://avantilipids.com/
