# Supplementary material for: Characterization of a Haematococcus pluvialis Diacylglycerol Acyltransferase 1 and Its Potential in Unsaturated Fatty Acid-Rich Triacylglycerol Production
Source: Front Plant Sci. 2021 Dec 7;12:771300. doi: 10.3389/fpls.2021.771300 (PMC8688921; doi:10.3389/fpls.2021.771300)
Supplement: Supplementary file 1 [file Data_Sheet_1.PDF]

Supplementary Table S1 | GenBank accession numbers for DGAT and ACTIN proteins used in this study. Cz, *Chromochloris zofingiensis*; No, *Nannochloropsis oceanica*; Cr, *Chlamydomonas reinhardtii*; Pt, *Phaeodactylum tricornutum*; Li, *Lobosphaera incise*; At, *Arabidopsis thaliana*; Gm, *Glycine max*; Zm, *Zea mays*; Sc, *Saccharomyces cerevisiae*.

Gene	GenBank No.	Gene	GenBank No.
CzDGAT1A	QBG05553.1	*CrDGAT1*	XP_001692975.1
CzDGAT1B	QBG05554.1	*CrDGAT2A*	AGO32156.1
CzDGAT2A	QBG05555.1	*CrDGAT2B*	AGO32157.1
CzDGAT2B	QBG05556.1	*CrDGAT2C*	AGO32158.1
CzDGAT2C	QBG05557.1	*CrDGAT2D*	AGO32159.1
CzDGAT2D	QBG05558.1	*CrDGAT2E*	XP_001701667.1
CzDGAT2E	QBG05559.1	*CrDGAT3*	XP_001691342.1
CzDGAT2F	QBG05560.1	*PtDGAT1*	ADY76581.1
CzDGAT2G	QBG05561.1	*PtDGAT2A*	AFQ23659.1
CzDGAT2H	QBG05562.1	*PtDGAT2B*	AFM37314.1
NoDGAT1A	ASL69957.1	*PtDGAT2C*	AFQ23660.1
NoDGAT1B	ASL69958.1	*PtDGAT2D*	AFQ23661.1
NoDGAT2A	ATB53137.1	*PtWSD*	XP_002180007.1
NoDGAT2B	ATB53138.1	*AtDGAT1*	CAB45373.1
NoDGAT2C	ATB53139.1	*AtDGAT2*	NP_566952.1
NoDGAT2D	ATB53140.1	*GmDGAT3*	XP_003542403.1
NoDGAT2E	ATB53141.1	*GmWSD*	XP_003552517.1
NoDGAT2F	ATB53142.1	*LiDGAT1*	MF576159
NoDGAT2G	ATB53143.1	*LiDGAT2.1*	MH290880
NoDGAT2H	ATB53144.1	*LiDGAT2.2*	MH290881
NoDGAT2I	ATB53145.1	*LiDGAT2.3*	MH290882
NoDGAT2J	ATB53146.1	*ZmDGAT3*	PWZ45621.1
NoDGAT2K	ATB53136.1	*HpDGAT1*	MT612720
ScACTIN	AAA34391.1	*CrACTIN*	D50838.1
AtACTIN	NP_190236.1		

Supplementary Table S2 | Primers used in this study.

Names	Forward primer (5'–3')	Reverse primer (5'–3')	Note
HpDGAT1-5-R1		CGCTGTGGTGTTGGCGGCGTTAAGC	5'-RACE
HpDGAT1-5-R2		ACCCAAGGGAGCTCTTGTCCTGAC	5'-RACE
HpDGAT1-3-F1	TGCCGGTGCACAAGTGGCTGCTG		3'-RACE
HpDGAT1-3-F2	TGTGTTCTTTGTGAGCGCTGTG		3'-RACE
HpDGAT1-ORF-F/R	ATGTGCGGCAGCAGCAGCAGCAGCA	TCAGATGACAATGCCGGAGCTTGTGTT	ORF clone
HpDGAT1-Q-F/R	GCAAGAGGCGACGGACAGGTA	TCGGGCAGCAAGGAGAAACG	qRT-PCR
HpACTIN-Q-F/R	AGCGGGAGATAGTGCGGGACA	ATGCCCACCGCCTCCATGC	qRT-PCR
HpDGAT1-YES-F/R	gaattcATGGCAAGCCTGCTGTCTTCAGAGCTC	agatctTCAGATGACAATGCCGGAGCTTGTGTT	Yeast
ScACTIN-Q-F/R	ACGTCGCCTTGGACTTCGAA	AGATGGAGCCAAAGCGGTGA	qRT-PCR
HpDGAT1-Cr-DB-F/R	cacgtgATGTGCGGCAGCAGCAGCAGCAGC	cgatcgTTAGATCACGATGCCGCTGCTGGTGTTCA	Algae
HpDGAT1-Cr-Q-F/R	GCACCATCTGCTGCCAGTTCG	GGGTCAGGTTCTCGGGGTAGG	qRT-PCR
CrACTIN-Q-F/R	GATGCCACGGGCGATGATTGA	CGTTGAGTACCCTCCCTGCGTTG	qRT-PCR
HpDGAT1-1303-F/R	ccatggATGGCAAGCCTGCTGTCTTCAGAGCTC	actagtTCAGATGACAATGCCGGAGCTTGTGTT	Plants
AtACTIN-Q-F/R	ATGACATGGAGAAGATCTGGCATCA	AGCCTGGATGGCAACATACATAGC	qRT-PCR

Sequences in lower-case letters indicate enzyme restriction sites.

A

Gene	Location	Score	E-value	Query	NCBI No.
***HpDGAT1* (partial)**	comp37256_c1_seq1_10	309	2e-084	*AtDGAT1*	AEC06882
***HpDGAT1* (partial)**	comp37256_c1_seq1_10	363	8e-101	*CvDGAT1*	ALP13865
***HpDGAT1* (partial)**	comp37256_c1_seq1_10	270	6e-073	*PtDGAT1*	ADY76581

B

>*HpDGAT1* (partial)

AGCCAGAGCAGCAGGCCTCCACCCAGCCCTGTGACCCAGCAGCAGCAGCAGCTTCAGCGGCGGCCACTCCACATATCGCGG
CCCGCAAGCCTGCTGTCTTCAGAGCTCATCCGGCTGCTCCATCAGAGCGGTGTGGTGAACCTGGTGTTCATGATCCTCGCGTCCGC
CAATTTCCGCCTCATCCTGGAGAACATGATGAAGTACGGGTTGCGCTTCAACCGTTTCTCCTTGCTGCCCGACGCCATCACTCCTT
GTGGCGACACGCCAGCTGGATCTGTGCTGATCCGATGTGGCCTTCTATTCGCTGAGCGGACTGGCCATCGAACCATTTGCTGTCAG
TTTGCTGGACATGGACGGTACCGAGGCAGCTGTCAGGACAAGAGCTCCCTTGGGTACATTCCAATGTACGGATCCCTAGCCAGGC
AATCAGCCAGGGGCGAGTGGGTGCTGTTCTGGCTTAACGCCGCCAACACCACAGCGGCTGTGGCCCTTCCCTGGGCTGTCATCTT
CACTACCAAGGCAGAGCCGCTGTCAGCTGCTGTGCTCATCTGCCTGGCTGTGGTGTTGTGGATGAAGCTCGTCAGCTTCCACCACT
GCTGCGCTGACCTGCGACAAGCCAGGCGCCAGGGGGAGGTGCGGCCAGGTGAGCGCGGCAGCCCAGGCTCGCCTGCGGAGTGGG
CCCTGCTGGCCTACCCTGAGAATCTCACACTCTCACACCTGGCCTACTTCCTGGCAGCTCCCACCCTGTCCTATCAGGTCAACTAC
CCGCGCACAGGCACCATCCGCAAGAAGTGGCTGGCCCGCCGGGTGGGAGAGCTGGTGGTCATGCTCACGGTGTTGTCCCTCATCG
TGGCGCAGTATGTGCAGCCGGCAGTTGCAAACTCCATGGGCCCACTCATGCAGATGGACTGGCTGCACATAGTGGAGCGGGTGCT
AAAGCTAGCGCTGCCCAACATGTACTGCTGGCTGGTCATGTTCTACTGCGTGTTCCACTTGTGGCTCAACATCCTTGCGGAGCTGC
TGAGGTTTGGTGACCGGGAGTTCTACAAGGACTGGTGGAACAGCAGCGATGTGGGCAGCTACTGGAAGCAATGGAACCTGCCGG
TGCACAAGTGGCTGCTGCGCCACGTCTACTTCCCCGCCCTGCGTCTGGGTTTGTCCAGGTGGCCGGCCATGATCCTTGTGTTCTTTG
TGAGCGCTGTGTTTCACGAGCTGGTGCTGGGCGTGCCCCTGCACATGGTGCGGCTGTGGGCCTTCAGCGGCATCATGCTGCAGGT
GCCGCTGGTCATCCTCACAGACTACGCCCGCAAGAAGCTGAACAGGGACGAAGCTGGCAACATCGTGTTCTGGCTCAGCTTCTGC
GTGGTGGGACAGCCGCTC

C

>HpDGAT1 (partial)

VTQQQQQQLQRRPLHISRPASLLSSELIRLLHQSGVVNLVFMILASANFRLILENMMKYGLRFNRFSLLPDAITPCGDTPAGSVLIRCGLL
FAERTGHRTICCQFAGHGRYRGSCQDKSSLGYIPMYGSLARQSARGEWVLFWLNAANTTAAVALPWAVIFTTKAEPLSAAVLICLAVV
LWMKLVSFHHCCADLRQARRQGEVRPGERGSPGSPAEWALLAYPENLTLSHLAYFLAAPTLSYQVNYPRTGTIRKKWLARRVGELVV
MLTVLSLIVAQYVQPAVANSMGPLMQMDWLHIVERVLKLALPNMYCWLVMFYCVFHLWLNILAELLRFGDREFYKDWWNSSDVGS
YWKQWNLPVHKWLLRHVYFPALRLGLSRWPAMILVFFVSAVFHELVLGVPLHMVRLWAFSGIMLQVPLVILTDYARKKLNRDEAGNI
VFWLSFCVVGQPL

D

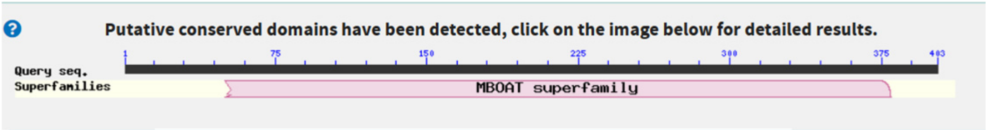

diacylgylcerol acyltransferase type-1 [Chlorella vulgaris]

Sequence ID: ALP13863.1 Length: 460 Number of Matches: 1

See 1 more title(s)

Range 1: 110 to 460 GenPept Graphics ▼ Next Match ▲ Previous Match

Score	Expect	Method	Identities	Positives	Gaps
387 bits(993)	3e-128	Compositional matrix adjust.	186/351(53%)	244/351(69%)	1/351(0%)

```
Query  46   QRRRKLARQSARGEWVLFWLNAANTTAAVALPWAVIFTTKAEPLSAAVLICLAVVLWMKL  105
            +  +  AR+++  E ++F LN  NTTAA+  P A+I  T AE +    L      VLW+KL
Sbjct  110  EMNKAAARRASATEHLVFVLNLLNTTAALLAPCAIILHTCAELMPGFALTMATCVLWLKL  169

Query  106  VSFHHCCADLRQARRQGEVRPGERGSPGSPAEWAL-LAYPENLTLSHLAYFLAAPTLSYQ  164
            VS+ H   D R ARR+GE RPGERGS   P       L YPEN+ L +LAYFLAAPTL YQ
Sbjct  170  VSYAHVNFDYRVARRRGETRPGERGSGQYPENVEQELRYPENIALGNLAYFLAAPTLCYQ  229

Query  165  VNYPRTGTIRKKWLARRVGELVVMLTVLSLIVAQYVQPAVANSMGPLMQMDWLHIVERVL  224
            + YPR+   R +W+ +++       L ++   +  QY+QP + NSM PL +MDWL ++ER+L
Sbjct  230  ITYPRSKRFRARWMVKKLFMFTGGLGLMLFVTEQYIQPTIDNSMRPLKEMDWLRMLERIL  289

Query  225  KLALPNMYCWLVMFYCVFHLWLNILAELLRFGDREFYKDWWNSSDVGSYWKQWNLPVHKW  284
            KL++P +Y WL MFY +F LWLNILAE+LRFGDREFYK+WWN++ VG YW+ WN PVHKW
Sbjct  290  KLSIPTLYWWLAMFYTLFDLWLNILAEVLRFGDREFYKEWWNATTVGEYWRLWNQPVHKW  349

Query  285  LLRHVYFPALRLGLSRWPAMILVFFVSAVFHELVLGVPLHMVRLWAFSGIMLQVPLVILT  344
            +LRHVYFP LR  + ++ A   VFF+SAVFHE+++GVPLHMVRLWAF G+M QVPL+I+T
Sbjct  350  MLRHVYFPLLRHKVPKFYAGFAVFFISAVFHEVLVGVPLHMVRLWAFWGLMAQVPLMIVT  409

Query  345  DYARKKLNRDEAGNIVFWLSFCVVGQPLTLLLYSHDYLYEVKPHLMATQAV  395
            ++ + KL  D  GNIVFW+SFC VGQPL + LY HD+L   K     T  +
Sbjct  410  EWLKNKLKNDRIGNIVFWISFCFVGQPLAMTLYYHDHLQGYKHPFQGTPII  460
```

Supplementary Figure S1 | The prediction of putative gene encoding HpDGAT1 in *H. pluvialis* based on transcription database. (A) The BLAST result, (B) The partial coding nucleotide sequences of putative *HpDGAT1*, (C) The partial coding protein sequences of putative HpDGAT1, (D) The NCBI blast identification of HpDGAT1.

A

```
   1 gatggaggagcgtccggtctagcacttttaatgccgtggttttgtagttattgccttgcactgggcgtttgagcacctgcgaaattgggccctggcatggcgtgcctgtgacgccgctgg
 120 agcgcgatgtgcggcagcagcagcagcagcacagttccagccgcaagaggcgacggacaggtagtgccagccctgcacctctgcagcagccagagcagcaggcctccacccagccctgtg
   1       M  C  G  S  S  S  S  S  T  V  P  A  A  R  G  D  G  Q  V  V  P  A  L  H  L  C  S  S  Q  S  S  R  P  P  P  S  P  V
 240 acccagcagcagcagcagcagcttcagcggcggccactccacatatcgcggcccgcaagcctgctgtcttcagagctcatccggctgctccatcagagcggtgtggtgaacctggtgttc
  39 T  Q  Q  Q  Q  Q  Q  L  Q  R  R  P  L  H  I  S  A  P  A  S  L  L  S  S  E  L  I  R  L  L  H  Q  S  G  V  V  N  L  V  F
 360 atgatcctcgcgtccgccaatttccgcctcatcctggagaacatgatgaagtacgggttgcgcttcaaccgtttctccttgctgcccgacgccatcactccttgtggcgacacgccagct
  79 M  I  L  A  S  A  N  F  R  L  I  L  E  N  M  M  K  Y  G  L  R  F  N  R  F  S  L  L  P  D  A  I  T  P  C  G  D  T  P  A
 480 ggatctgtgctgatccgatgtggccttctattcgctgagcggactggccatcgaaccatttgctgtcagtttgctggacatggacggtaccgaggcagctgtcaggacaagagctccctt
 119 G  S  V  L  I  R  C  G  L  L  F  A  E  R  T  G  H  R  T  I  C  C  Q  F  A  G  H  G  R  Y  R  G  S  C  Q  D  K  S  S  L
 600 gggtacattccaatgtacggatccctagccaggcaatcagccaggggcgagtgggtgctgttctggcttaacgccgccaacaccacagcggctgtggcccttccctgggctgtcatcttc
 159 G  Y  I  L  M  Y  A  S  L  A  R  Q  S  A  R  G  E  W  V  L  F  W  L  N  A  A  N  T  T  A  A  V  A  L  P  W  A  V  I  F
 720 actaccaaggcagagccgctgtcagctgctgtgctcatctgcctggctgtggtgttgtggatgaagctcgtcagcttccaccactgctgcgctgacctgcgacaagccaggcgccagggg
 199 T  T  K  A  E  P  L  S  A  A  V  L  I  C  L  A  V  V  L  W  M  K  L  V  S  F  H  H  C  C  A  D  L  R  Q  A  R  R  Q  G
 840 gaggtgcggccaggtgagcgcggcagcccaggctcgcctgcggagtgggccctgctggcctaccctgagaatctcacactctcacacctggcctacttcctggcagctcccaccctgtcc
 239 E  V  R  P  G  E  R  G  S  P  G  S  P  A  E  W  A  L  L  A  Y  P  E  N  L  T  L  S  H  L  A  Y  F  L  A  A  P  T  L  S
 960 tatcaggtcaactacccgcgcacaggcaccatccgcaagaagtggctggcccgccgggtgggagagctggtggtcatgctcacggtgttgtccctcatcgtggcgcagtatgtgcagccg
 279 Y  Q  V  N  Y  P  R  T  G  T  I  R  K  K  W  L  A  R  R  V  G  E  L  V  V  M  L  T  V  L  S  L  I  V  A  Q  Y  V  Q  P
1080 gcagttgcaaactccatgggcccactcatgcagatggactggctgcacatagtggagcgggtgctaaagctagcgctgcccaacatgtactgctggctggtcatgttctactgcgtgttc
 319 A  V  A  N  S  M  G  P  L  M  Q  M  D  W  L  H  I  V  E  R  V  L  K  L  A  L  P  N  M  Y  C  W  L  V  M  F  Y  C  V  F
1200 cacttgtggctcaacatccttgcggagctgctgaggtttggtgaccgggagttctacaaggactggtggaacagcagcgatgtgggcagctactggaagcaatggaacctgccggtgcac
 359 H  L  W  L  N  I  L  A  E  L  L  R  F  G  D  R  E  F  Y  K  D  W  W  N  S  S  D  V  G  S  Y  W  K  Q  W  N  L  P  V  H
1320 aagtggctgctgcgccacgtctacttccccgccctgcgtctgggtttgtccaggtggccggccatgatccttgtgttctttgtgagcgctgtgtttcacgagctggtgctgggcgtgccc
 399 K  W  L  L  R  H  V  Y  F  P  A  L  R  L  G  L  S  R  W  P  A  M  I  L  V  F  F  V  S  A  V  F  H  E  L  V  L  G  V  P
1440 ctgcacatggtgcggctgtgggccttcagcggcatcatgctgcaggtgccgctggtcatcctcacagactacgcccgcaagaagctgaacagggacgaagctggcaacatcgtgttctgg
 439 L  H  M  V  R  L  W  A  F  S  G  I  M  L  Q  V  P  L  V  I  L  T  D  Y  A  R  K  K  L  N  R  D  E  A  G  N  I  V  F  W
1560 ctcagcttctgcgtggtgggacagccgctcacgctgctcctctactcccatgactacctctatgaggtcaagcctcacctgatggccactcaggctgtcaacacaagctccggcattgtc
 479 L  S  F  C  V  V  G  Q  P  L  T  L  L  L  Y  S  H  D  Y  L  Y  E  V  K  P  H  L  M  A  T  Q  A  V  N  T  S  S  G  I  V
1680 atctgagtggctcatgacatgacgccaatggtgccagtggtctggccgcaggagttctgcgaggcaatggccttggtcttctcctctgtctcaataactcaatgtggaacatcttggaga
 519 I  *
1800 aaaaaaaaaaa
```

B

```
### chlorop v1.1 prediction results ##########################
Number of query sequences: 1

Name                Length      Score  cTP       CS-    cTP-
                                                score  length
-------------------------------------------------------------
HpDGAT1                519      0.572   Y       8.631     56
-------------------------------------------------------------
```

C

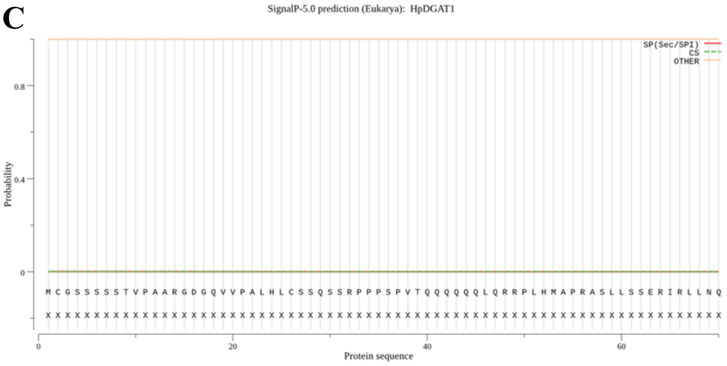

D

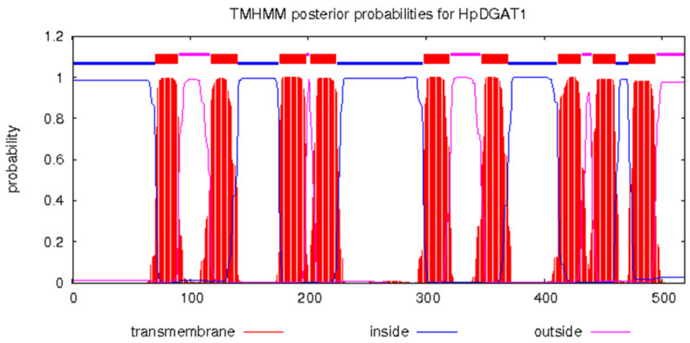

E

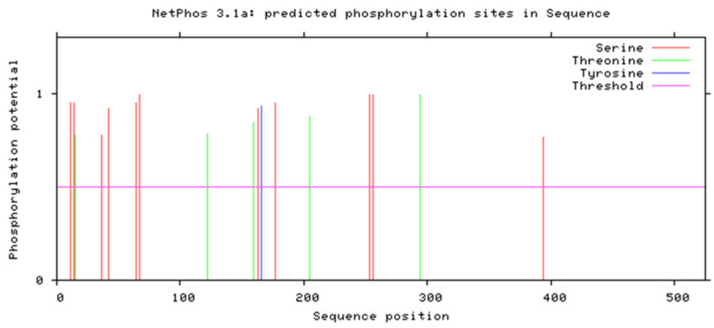

Supplementary Figure S2 | The basic physical and chemical properties of HpDGAT1. (A) The full-length nucleotide and deduced amino acids sequence of HpDGAT1.The letters underlined indicate the start codon (ATG) and the stop codon (TAA). The conserved motifs (CMs) were indicated by red color. The predicted CTP sequences were indicated by yellow shadow. The conserved amino acid residues involved in activity were indicated by blue color. (B) The sub-cellular location analysis. (C) The SignalP analysis. (D) The trans-membrane regions analysis. (E) The phosphorylation sites analysis.

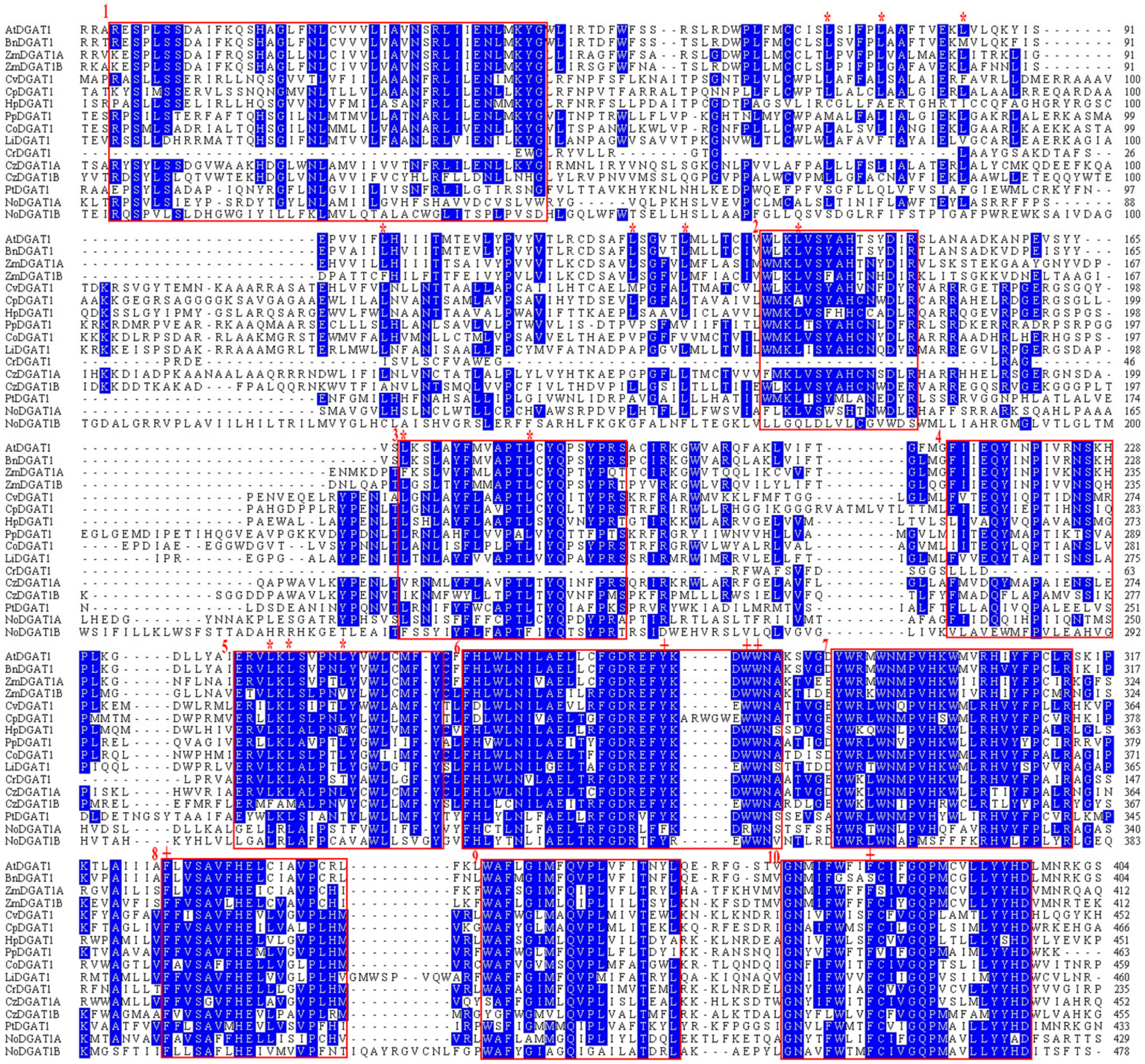

Supplementary Figure S3 | Protein sequence alignment of putative HpDGAT1 and other annotated DGAT1s from plants and microalgae. Protein sequences used in this study were listed in in Table S1. Red box indicates the key conserved motifs and red asterisk and pluses indicate the key amino acid residues involved in enzyme activity.

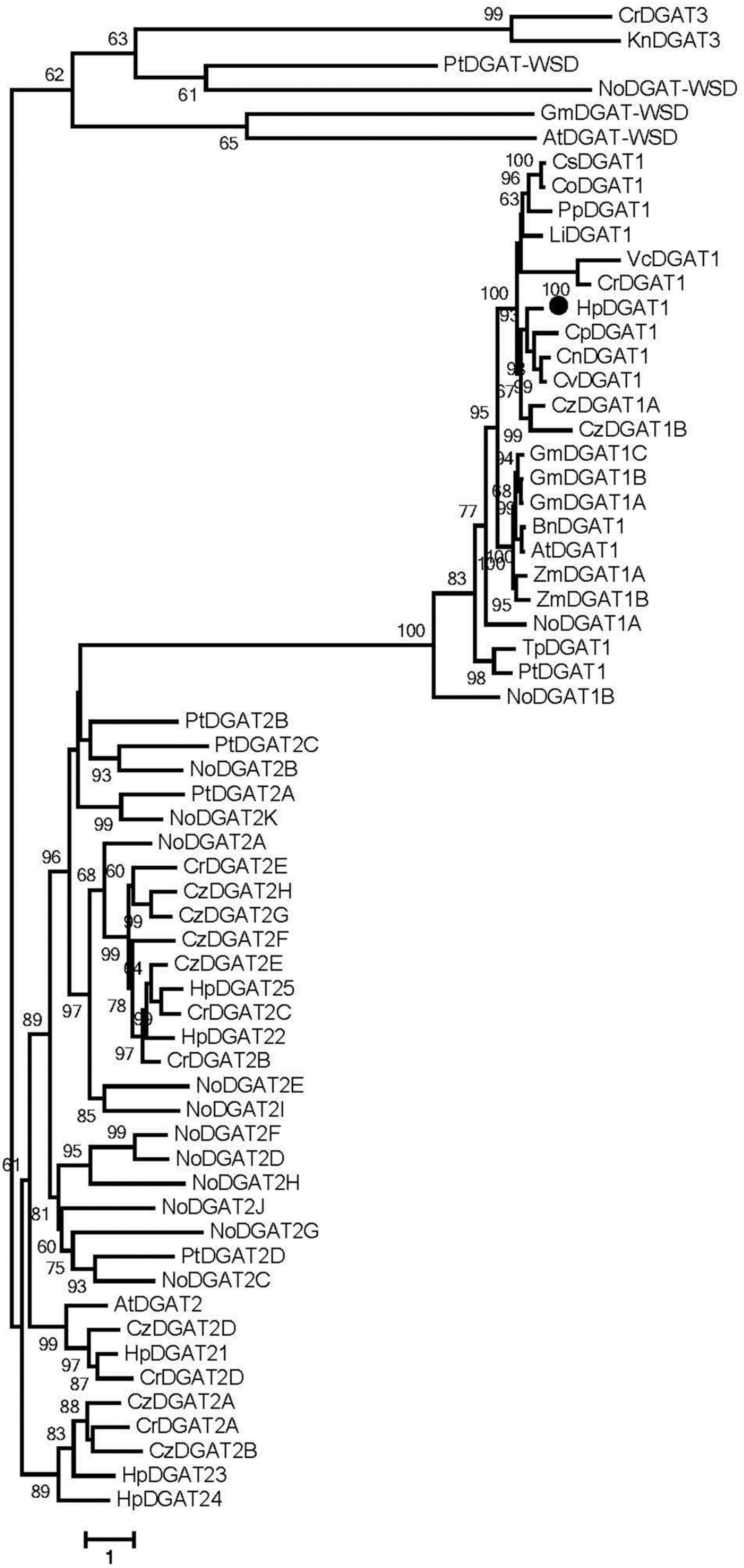

Supplementary Figure S4 | Phylogenetic analysis of HpDGAT1 and other annotated DGATs from higher plants and microalgae. Protein sequences used in this study were listed in Supplementary Table S1.

>*HpDGAT1* (original sequence)
ATGTGCGGCAGCAGCAGCAGCAGCACAGTTCCAGCCGCAAGAGGCGACGGACAGGTAGTGCCAGCCCTGCACC
TCTGCAGCAGCCAGAGCAGCAGGCCTCCACCCAGCCCTGTGACCCAGCAGCAGCAGCAGCAGCTTCAGCGGCGG
CCACTCCACATATCGCGGCCCGCAAGCCTGCTGTCTTCAGAGCTCATCCGGCTGCTCCATCAGAGCGGTGTGGTG
AACCTGGTGTTCATGATCCTCGCGTCCGCCAATTTCCGCCTCATCCTGGAGAACATGATGAAGTACGGGTTGCGC
TTCAACCGTTTCTCCTTGCTGCCCGACGCCATCACTCCTTGTGGCGACACGCCAGCTGGATCTGTGCTGATCCGAT
GTGGCCTTCTATTCGCTGAGCGGACTGGCCATCGAACCATTTGCTGTCAGTTTGCTGGACATGGACGGTACCGAG
GCAGCTGTCAGGACAAGAGCTCCCTTGGGTACATTCCAATGTACGGATCCCTAGCCAGGCAATCAGCCAGGGGC
GAGTGGGTGCTGTTCTGGCTTAACGCCGCCAACACCACAGCGGCTGTGGCCCTTCCCTGGGCTGTCATCTTCACT
ACCAAGGCAGAGCCGCTGTCAGCTGCTGTGCTCATCTGCCTGGCTGTGGTGTTGTGGATGAAGCTCGTCAGCTTC
CACCACTGCTGCGCTGACCTGCGACAAGCCAGGCGCCAGGGGGAGGTGCGGCCAGGTGAGCGCGGCAGCCCAG
GCTCGCCTGCGGAGTGGGCCCTGCTGGCCTACCCTGAGAATCTCACACTCTCACACCTGGCCTACTTCCTGGCAG
CTCCCACCCTGTCCTATCAGGTCAACTACCCGCGCACAGGCACCATCCGCAAGAAGTGGCTGGCCCGCCGGGTG
GGAGAGCTGGTGGTCATGCTCACGGTGTTGTCCCTCATCGTGGCGCAGTATGTGCAGCCGGCAGTTGCAAACTC
CATGGGCCCACTCATGCAGATGGACTGGCTGCACATAGTGGAGCGGGTGCTAAAGCTAGCGCTGCCCAACATGT
ACTGCTGGCTGGTCATGTTCTACTGCGTGTTCCACTTGTGGCTCAACATCCTTGCGGAGCTGCTGAGGTTTGGTG
ACCGGGAGTTCTACAAGGACTGGTGGAACAGCAGCGATGTGGGCAGCTACTGGAAGCAATGGAACCTGCCGGT
GCACAAGTGGCTGCTGCGCCACGTCTACTTCCCCGCCCTGCGTCTGGGTTTGTCCAGGTGGCCGGCCATGATCCT
TGTGTTCTTTGTGAGCGCTGTGTTTCACGAGCTGGTGCTGGGCGTGCCCCTGCACATGGTGCGGCTGTGGGCCTT
CAGCGGCATCATGCTGCAGGTGCCGCTGGTCATCCTCACAGACTACGCCCGCAAGAAGCTGAACAGGGACGAA
GCTGGCAACATCGTGTTCTGGCTCAGCTTCTGCGTGGTGGGACAGCCGCTCACGCTGCTCCTCTACTCCCATGAC
TACCTCTATGAGGTCAAGCCTCACCTGATGGCCACTCAGGCTGTCAACACAAGCTCCGGCATTGTCATCTGA

>*HpDGAT1-Cr* (new sequence after codon optimization for *Chlamydomonas reinhardtii*)
ATGTGCGGCAGCAGCAGCAGCAGCACCGTGCCCGCCGCCCGCGGCGACGGCCAGGTGGTGCCCGCCCTGCACCT
GTGCAGCAGCCAGAGCAGCCGCCCCCCCCCCAGCCCCGTGACCCAGCAGCAGCAGCAGCAGCTGCAGCGCCGCC
CCCTGCACATCAGCCGCCCCGCCAGCCTGCTGAGCAGCGAGCTGATCCGCCTGCTGCACCAGAGCGGCGTGGTG
AACCTGGTGTTCATGATCCTGGCCAGCGCCAACTTCCGCCTGATCCTGGAGAACATGATGAAGTACGGCCTGCG
CTTCAACCGCTTCAGCCTGCTGCCCGACGCCATCACCCCCTGCGGCGACACCCCCGCCGGCAGCGTGCTGATCCG
CTGCGGCCTGCTGTTCGCCGAGCGCACCGGCCACCGCACCATCTGCTGCCAGTTCGCCGGCCACGGCCGCTACCG
CGGCAGCTGCCAGGACAAGAGCAGCCTGGGCTACATCCCCATGTACGGCAGCCTGGCCCGCCAGAGCGCCCGCG
GCGAGTGGGTGCTGTTCTGGCTGAACGCCGCCAACACCACCGCCGCCGTGGCCCTGCCCTGGGCCGTGATCTTCA
CCACCAAGGCCGAGCCCCTGAGCGCCGCCGTGCTGATCTGCCTGGCCGTGGTGCTGTGGATGAAGCTGGTGAGC
TTCCACCACTGCTGCGCCGACCTGCGCCAGGCCCGCCGCCAGGGCGAGGTGCGCCCCGGCGAGCGCGGCAGCCC
CGGCAGCCCCGCCGAGTGGGCCCTGCTGGCCTACCCCGAGAACCTGACCCTGAGCCACCTGGCCTACTTCCTGG
CCGCCCCCACCCTGAGCTACCAGGTGAACTACCCCCGCACCGGCACCATCCGCAAGAAGTGGCTGGCCCGCCGC
GTGGGCGAGCTGGTGGTGATGCTGACCGTGCTGAGCCTGATCGTGGCCCAGTACGTGCAGCCCGCCGTGGCCAA
CAGCATGGGCCCCCTGATGCAGATGGACTGGCTGCACATCGTGGAGCGCGTGCTGAAGCTGGCCCTGCCCAACA
TGTACTGCTGGCTGGTGATGTTCTACTGCGTGTTCCACCTGTGGCTGAACATCCTGGCCGAGCTGCTGCGCTTCG
GCGACCGCGAGTTCTACAAGGACTGGTGGAACAGCAGCGACGTGGGCAGCTACTGGAAGCAGTGGAACCTGCC
CGTGCACAAGTGGCTGCTGCGCCACGTCTACTTCCCCGCCCTGCGCCTGGGCCTGAGCCGCTGGCCCGCCATGAT
CCTGGTGTTCTTCGTGAGCGCCGTGTTCCACGAGCTGGTGCTGGGCGTGCCCCTGCACATGGTGCGCCTGTGGGC
CTTCAGCGGCATCATGCTGCAGGTGCCCCTGGTGATCCTGACCGACTACGCCCGCAAGAAGCTGAACCGCGACG
AGGCCGGCAACATCGTGTTCTGGCTGAGCTTCTGCGTGGTGGGCCAGCCCCTGACCCTGCTGCTGTACAGCCACG
ACTACCTGTACGAGGTGAAGCCCCACCTGATGGCCACCCAGGCCGTGAACACCAGCAGCGGCATCGTGATCTA
A

Supplementary Figure S5 | The original sequence of *HpDGAT1* in *Haematococcus pluvialis* and the new sequence of *HpDGAT1-Cr* after codon optimization for *Chlamydomonas reinhardtii*.

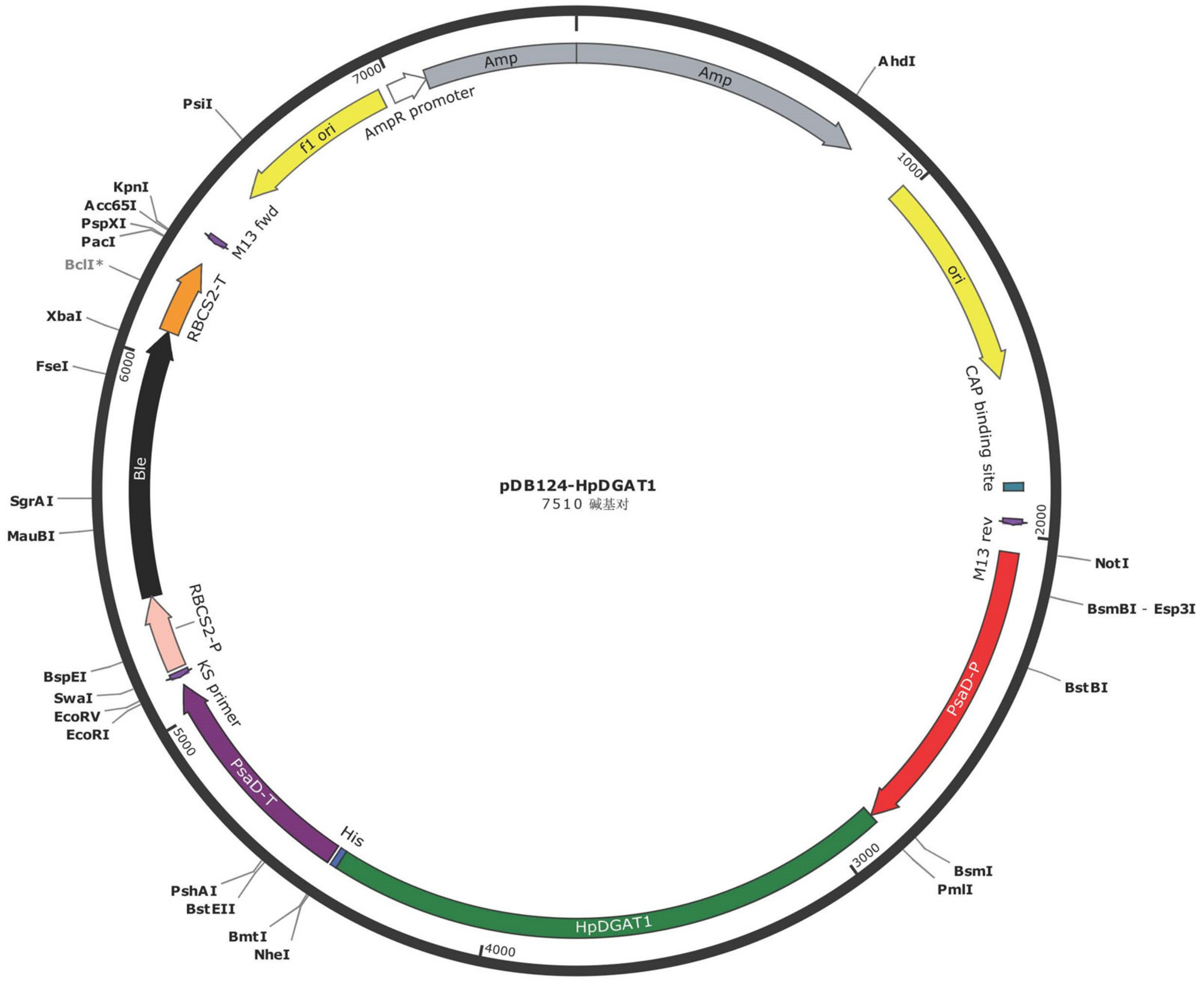

Supplementary Figure S6 |A schematic map of the pDB124-*HpDGAT1* plasmid. This vector contained an expression cassette of the *HpDGAT1* gene under the control of the endogenous and characterized *PsaD* promoter and *PsaD* terminator, an expression cassette of the *Ble* gene controlled by the endogenous and characterized *RBCS2* promoter and *RBCS2* terminator, and an expression cassette of the *Amp* resistance gene, which conferred resistance to ampicillin.

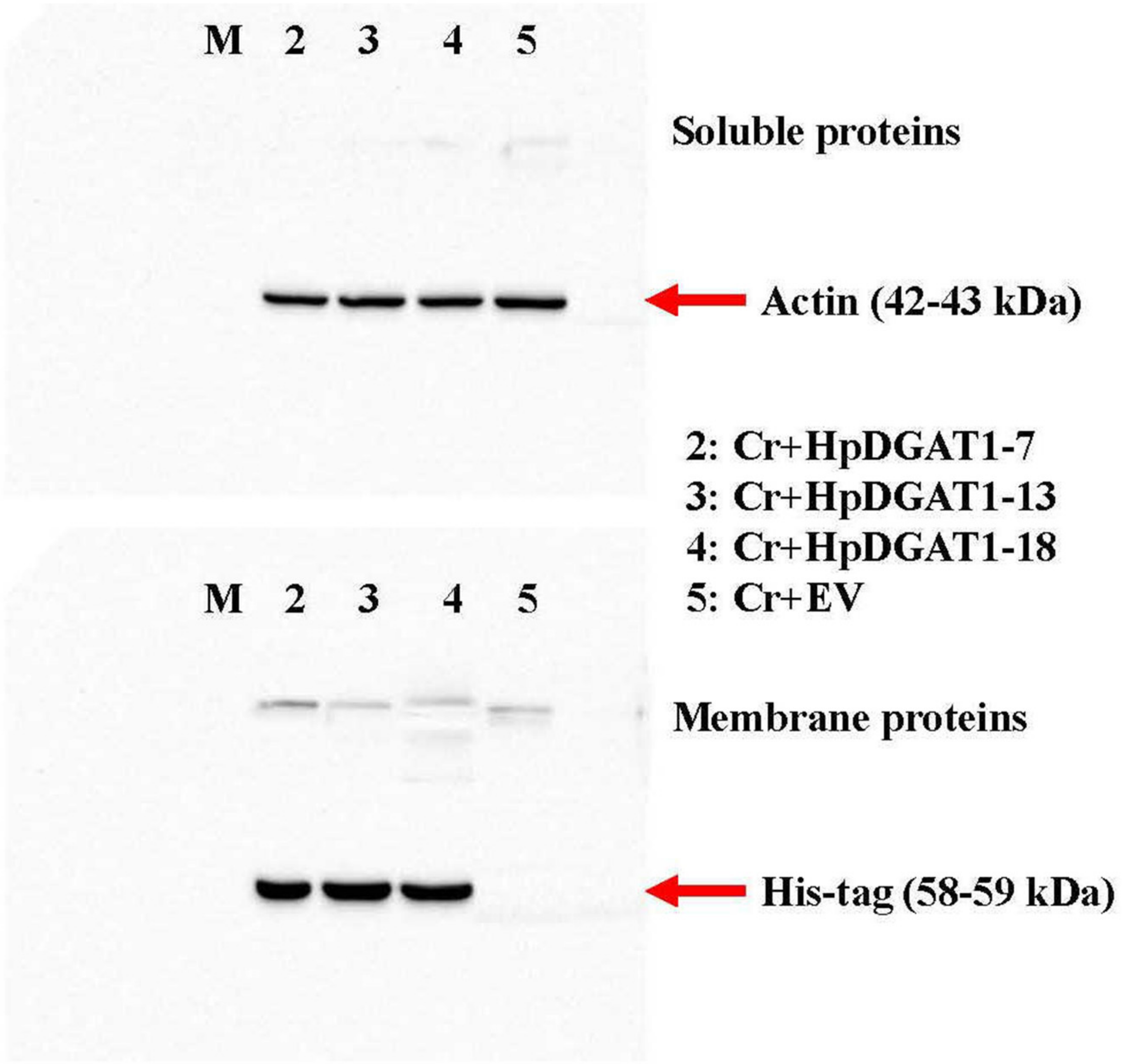

Supplementary Figure S7 | Western blotting of HpDGAT1-6-His tag fusion protein with His-tag antibodies. Soluble and membrane proteins were separated and used for blotting. Actin which was known soluble protein was used as controls.
